# Eremophilane- and Acorane-Type Sesquiterpenes from the Deep-Sea Cold-Seep-Derived Fungus *Furcasterigmium furcatum* CS-280 Cultured in the Presence of Autoclaved *Pseudomonas aeruginosa* QDIO-4

**DOI:** 10.3390/md22120574

**Published:** 2024-12-22

**Authors:** Xiao-Dan Chen, Xin Li, Xiao-Ming Li, Sui-Qun Yang, Bin-Gui Wang

**Affiliations:** 1 CAS and Shandong Province Key Laboratory of Experimental Marine Biology, Institute of Oceanology, Chinese Academy of Sciences, Nanhai Road 7, Qingdao 266071, China; chenxiaodan@qdio.ac.cn (X.-D.C.); lixin@qdio.ac.cn (X.L.); lixmqd@qdio.ac.cn (X.-M.L.); 2 University of Chinese Academy of Sciences, Yuquan Road 19A, Beijing 100049, China; 3 Laboratory for Marine Biology and Biotechnology, Qingdao Marine Science and Technology Center, Wenhai Road 1, Qingdao 266237, China

**Keywords:** eremophilane, acorane, sesquiterpenes, cold-seep derived fungus, *Furcasterigmium furcatum*

## Abstract

Six new sesquiterpenes, including four eremophilane derivatives fureremophilanes A–D (**1**–**4**) and two acorane analogues furacoranes A and B (**5** and **6**), were characterized from the culture extract of the cold-seep derived fungus *Furcasterigmium furcatum* CS-280 co-cultured with autoclaved *Pseudomonas aeruginosa* QDIO-4. All the six compounds were highly oxygenated especially **2** and **3** with infrequent epoxyethane and tetrahydrofuran ring systems. The structures of **1**–**6** were established on the basis of detailed interpretation of 1D and 2D NMR and MS data. Their relative and absolute configurations were assigned by a combination of NOESY and single crystal X-ray crystallographic analysis, and by time-dependent density functional (TDDFT) ECD calculations as well. All compounds were tested the anti-inflammatory activity against human COX-2 protein, among which, compounds **2** and **3** displayed activities with IC_50_ values 123.00 µM and 93.45 µM, respectively. The interaction mechanism was interpreted by molecular docking.

## 1. Introduction

The harsh environment in the deep sea forces the benthic microbes to develop sophisticated molecular adaptations in their primary and secondary metabolic pathways [1]. Cold seep is one of the most unique and extraordinary ecosystems in the deep sea. The microorganisms inhabiting in the cold seep environment were able to produce unconventional secondary metabolites with diverse and abundant biosynthetic gene clusters [2,3]. Eremophilane and acorane are two classes of sesquiterpenes with diverse structures, and are widely produced by various microorganisms, especially by fungal species [4]. These metabolites were reported to exhibit multiple biological activities, such as antibacterial [5], anti-fungal [6,7], cytotoxic [8], and anti-inflammatory [9] activities.

**Figure 1:**
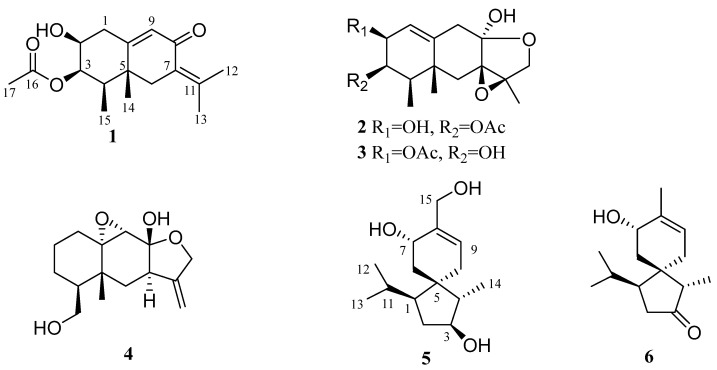
Chemical Structures of Compounds **1**–**6**.

We previously investigated the deep-sea cold-seep-derived fungus *Furcasterigmium furcatum* CS-280 and characterized several polyketide and ascochlorin derivatives with antibacterial activities from the rice solid culture of the fungus [10]. Co-cultivation was proved as an effective method to activate microbial biosynthetic genes which were silent under standard laboratory culture conditions [11,12,13]. There are several reports on co-cultivation experiments of fungi and heat-killed bacteria, leading to the discovery of new secondary metabolites, such as a series of sugar alcohol-conjugated acyclic sesquiterpenes and 16-residue peptaibols from the sponge-derived fungus *Acremonium* sp. cultivated with heat-killed *Pseudomonas aeruginosa* [14,15]. In the present report, the fungus *F. furcatum* CS-280 was co-cocultured with autoclaved *Pseudomonas aeruginosa* QDIO-4 for chemical investigation. As a result, six new sesquiterpenes, including four eremophilane derivatives fureremophilanes A–D (**1**–**4**) and two acorane analogues furacoranes A and B (**5** and **6**), were characterized (Figure 1). This paper describes the isolation, structure determination, stereochemical assignment, and bioactivity evaluation of the isolated compounds.

## 2. Results and Discussion

### 2.1. Structure Elucidation

**Figure 2:**
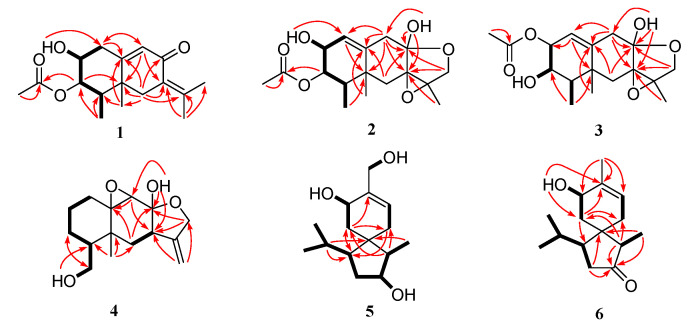
Key HMBC (arrows) and COSY (bold lines) correlations for compounds **1**–**6**.

**Figure 3:**
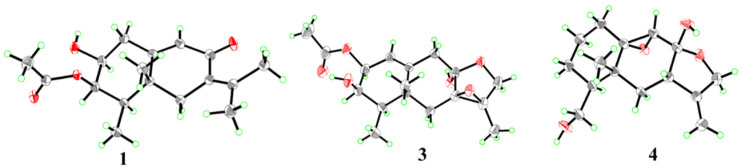
X-ray crystallographic structures of compounds **1**, **3**, and **4**.

Compound **1** was acquired as colorless crystals. Its chemical formula was assigned as C_17_H_24_O_4_ on the basis of the positive HRESIMS data at *m*/*z* 293.1751 (calculated for C_17_H_25_O_4_, [M + H]^+^, 293.1747), requiring six degrees of unsaturation. The ^1^H and ^13^C NMR data revealed the presence of five methyl groups, two sp^3^ methylenes, one sp^2^ and three sp^3^ methines (with two oxygenated), and six nonprotonated (with one sp^3^ and five sp^2^) carbons, along with an exchangeable proton (*δ*_H_ 5.05) (Table 1 and Table 2). Detailed analysis of the NMR data revealed that the structure of **1** was similar to (3*S*)-3-acetoxyeremophil-1(2),7(11),9(10)-trien-8-one, a known eremophilane sesquiterpene isolated from *Penicillium roqueforti* DAOM 232127, a fungus obtained from contaminated silage in Québec [16]. However, signals for two olefinic methines CH-1 (*δ*_H_ 5.79 and *δ*_C_ 130.75) and CH-2 (*δ*_H_ 5.93 and *δ*_C_ 131.04) in the NMR spectra of the known compound were absent, while resonances for a sp^3^ methylene CH_2_-1 (*δ*_H_ 2.55/2.30 and *δ*_C_ 35.8) and an oxygenated methine CH-2 (*δ*_H_ 3.64 and *δ*_C_ 68.5) were observed in the NMR spectra of **1** (Table 1 and Table 2), which indicated that the *Δ*^1(2)^-double bond of the known compound was reduced in compound **1**. Combined with the ^1^H-^1^H COSY correlations from H-2 to H_2_-1 and H-3 and to the proton of OH-2, and the key HMBC correlations from the proton of OH-2 to C-1 and from H_2_-1 to C-9 and C-10 (Figure 2), the planar structure of **1** was established. Due to absence of some diagnostic NOE correlations, its relative configuration could not be precisely assigned by NOESY experiment. The suitable crystals of **1** were obtained by slow evaporation of a mixed solvents (MeOH and H_2_O), allowing for the X-ray crystallographic analysis using Cu Kα radiation. The Flack parameter 0.09(5) permitted establishment of the absolute configurations as 2*S*, 3*R*, 4*R*, and 5*R* (Figure 3). Overall, the structure of **1** was determined and was given the trivial name fureremophilane A.

It should be mentioned that searching the structure of compound **1** in CAS SciFinder database gave a result showing “a compound” (CAS number 1027589-47-4) has 100% similarity to compound **1**. However, the structure of “as drawn” compound in the database has a double bond at *Δ*^11(12)^, which was different from that of compound **1** (with a double bond at *Δ*^7(11)^), and no reference describing the structure and property for the “compound” (with CAS number 1027589-47-4) could be found.

Compound **2** was isolated as yellow oil, of which the chemical formula was established as C_17_H_24_O_6_ by HRESIMS data. The ^1^H and ^13^C NMR data revealed the presence of four methyls, three methylenes, four methines, and six non-protonated carbons (Table 1 and Table 2). Discreet analyses of its NMR data revealed compound **2** had close relationship to 5-chloro-6-hydroxy-3a-methoxy-1a,8,8a-trimethyl-1a,2,3a,5,7,8,8a,9-octahydro-6*H*-naphtho [2,3-b]oxireno [2,3-c]furan-7-yl acetate, a chlorinated eremophilane sesquiterpene isolated from the fungal strain *Penicillium* sp. PR19N-1, which was obtained from antarctic deep-sea [17]. However, there is no chlorine substitution in compound **2**, as indicated by the HRESIMS data. The HMBC correlations from the exchangeable proton (*δ*_H_ 5.82, 8-OH) to C-7, C-8, and C-9 could illustrate that the methoxy group at C-8 in the reported compound also dismissed in **2**. On the other hand, the double bond in compound **2** located at *Δ*^1(10)^, as evidenced by the COSY correlations from the olefinic proton H-1 to the oxymehine proton H-2 as well as by the HMBC correlations from H-9 to C-1 and C-10 and from the proton of 2-OH to C-1 (Figure 2). The planar structure of compound **2** was thus determined (Figure 1).

Compound **3** was isolated as colorless crystals and has same molecular formula as that of compound **2**. The 1D and 2D NMR data revealed that compound **3** was reminiscent to compound **2** (Table 1 and Table 2). Supported by the COSY correlations from H-3 to the proton of 3-OH and H-2, by the HMBC correlation from H_3_-14 to C-3, and by the changes of chemical shifts of C-1, C-2, and C-3, the acetoxyl unit in **3** was supposed to be linked at C-2 instead of C-3 as that in **2** (Figure 2).

**Figure 4:**
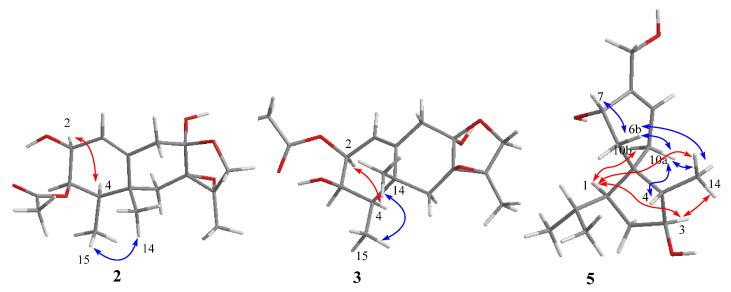
Key NOE correlations observed for compounds **2**, **3**, and **5** (red and blue arrows represent α- and β-orientations, respectively).

The relative configurations at C-2, C-4, and C-5 of compounds **2** and **3** were established by NOESY cross-peaks from H_3_-14 to H_3_-15, from H-2 to H-4 (Figure 4). The small coupling constant between H-3 and H-4 (^3^*J*_H-3, H-4_ = 2.2 Hz) in **3** assigned cofacial relationship of these two protons [17], while the relative configuration of hydroxy unit at C-8 and the epoxide group at C-7/C-11 were deduced as *α* and *β*-orientations, respectively, from the prospect of biosynthesis [17]. Single-crystal X-ray diffraction experiment of compound **3** was carried out to further confirm the relative configurations, and the Flack parameter 0.08(4) unambiguously assigned its absolute configurations as 2*S*, 3*R*, 4*R*, 5*R*, 7*R*, 8*R*, and 11*R* (Figure 3). The configurations of compounds **2** and **3** were considered consistent from the biosynthetic point of view. Compounds **2** and **3** were named as fureremophilanes B and C, respectively.

Compound **4** was obtained as colorless crystals and its HRESIMS data gave the molecular formula C_15_H_22_O_4_. The ^1^H and ^13^C NMR data resembled those of cryptosphaerolide, an ester-substituted (at C-1) eremophilane sesquiterpenoid characterized from the marine-sourced ascomycete fungal strain *Cryptosphaeria* sp. CNL-523 [18]. Detailed analysis of the NMR data of **4** showed that the ester moiety at C-1 in cryptosphaerolide is not present in compound **4**. Instead, C-1 is a non-oxygenated methylene and a hydroxy group was linked directly to C-15 in compound **4**, as verified by key ^1^H-^1^H COSY correlations from H_2_-2 to H_2_-1 and H_2_-3, from H-4 to H_2_-3 and H_2_-15, and from H_2_-15 to the proton of 15-OH (Figure 2). Single-crystal X-ray diffraction experiment not only confirmed the structure of compound **4** but also established its absolute configuration as 4*S*, 5*R*, 7*R*, 8*R*, 9*S*, and 10*S* (Figure 3). Thus, compound **4** was assigned as a new eremophilane derivative and named as fureremophilane D.

Furacorane A (**5**), obtained as yellowish oil, assigned the molecular formula C_15_H_26_O_3_ on the basis of HRESIMS data, indicating three degrees of unsaturation. Its ^13^C NMR data (Table 3) disclosed an acorane skeleton, which was similar to daphneaine C [19]. The presence of cyclopentane moiety of **5** was assigned by COSY correlations (measured in DMSO-*d*_6_) from H-1 through H_2_-2, H-3, and H-4 to H_3_-15, while the correlations from H-11 to H-1, H-12, and H-13 verified the presence of an isopropyl unit at C-1 (Figure 2). The COSY correlations from H-3 to 3-OH indicated the hydroxyl attached to C-3 in **5** instead of C-2 in daphneaine C. In addition, the methyl group at C-8 (CH_3_-15) in daphneaine C was replaced by a hydroxymethyl unit in compound **5,** as supported by the chemical shift of C-15 moving downfield significantly to *δ*_C_ 61.5 (C-15), along with the observed COSY from the proton of 15-OH to H_2_-15 (Figure 2) and the long distance COSY from H_2_-15 to H-9 (allylic coupling, ^4^*J*) and from H-7 and H_2_-15 to H-10a and H-10b (high allylic coupling, ^5^*J*) (Figure S39). For the severe overlapping of some signals, the ^1^H NMR, HSQC and NOESY spectra of compound **5** were re-measured in CDCl_3_ (Figures S41–S43). The NOE correlations from H-1 to H-3 and H_3_-14 and from H-3 to H_3_-14 implied their cofacial orientation (Figure 4). H-10a (*δ*_H_ 2.06), H-6b (*δ*_H_ 1.20), and H-7 were assigned cofacial, as determined by NOE correlations from H-10a to H-6b and from H-6b to H-7. Thus, the relative configurations at cyclopentane unit were established. NOE correlations from H-10a to H-4 and H_3_-14, from H-10b to H-1, and from H-6b to H_3_-14 revealed the H-10a, H-4, H_3_-14, H-6b and H-7 located on the same side of the cyclopentane unit, while H-10b and H-1 were on the other side. The relative configuration of **5** was thus established as shown (Figure 4).

To determine the absolute configuration, the specific rotation (SR) at the CAM-B3LYP/TZVP level was calculated (Tables S1–S3 in the Supplementary Data). The calculated SR value [α]_D_ –22.6 for (1*S*,3*S*,4*S*,5*R*,7*S*)-**5** was in agreement with the experimental value of **5** ${\left[\alpha \right]}_{D}^{25}$ –18.2 (*c* 0.11, MeOH), which allowed the assignment of the absolute configuration of **5** as 1*S*, 3*S*, 4*S*, 5*R*, and 7*S*.

**Figure 5:**
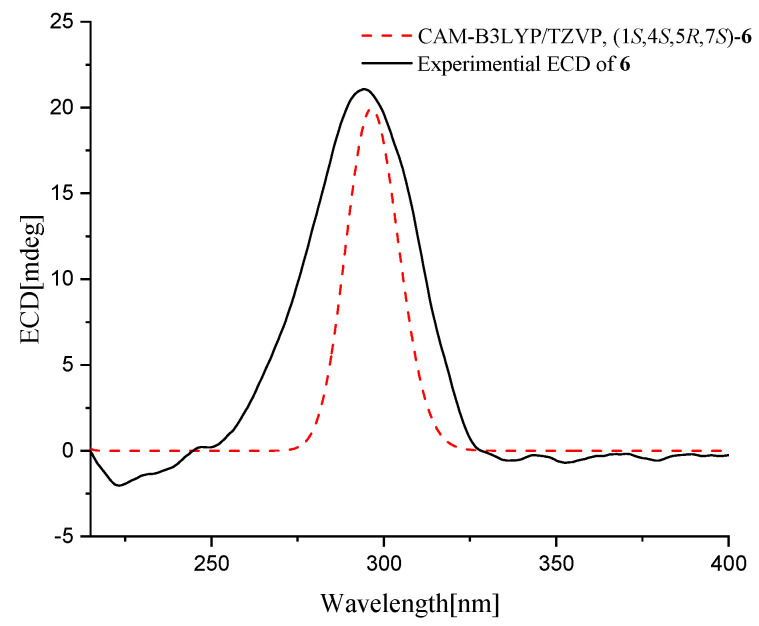
Experimental (black) and calculated (red) ECD spectra of compound **6**.

Furacorane B (**6**) was also obtained as yellowish oil. Its molecular formula was determined as C_15_H_24_O_2_ on the basis of (+)-HRESIMS data, with one O-atom less and one more degree of unsaturation than that of **5**. In the ^13^C NMR of **6**, a resonance corresponding to a ketonic carbonyl at *δ*_C_ 220.5 (C-3) was observed, and the location of this carbonyl carbon was determined at C-3 by the detected HMBC correlations from H-2, H-4, and H_3_-14 to C-3 (Figure 2). The chemical shift of C-15 shifted from *δ*_C_ 61.5 in **5** to the upfield at *δ*_C_ 19.7 in **6**, revealing the absence of -OH at C-15 (Table 3). Unfortunately, it is infeasible to determine the relative configuration by NOESY spectrum due to the special spiral ring system and the severe signal overlapping. The relative configuration of **6** was established the same as **5** owing to the same biosynthetic pathway. To further verify the absolute configuration of **6**, ECD calculation was performed at the CAM-B3LYP/TZVP level in Gaussian 09. The experimental curve matched well with that of the calculated ECD spectrum for 1*S*, 4*S*, 5*R*, 7*S*-**6** (Figure 5), both with positive Cotton effects around 295 nm. Thus, the absolute configuration of **6** was finally assigned as 1*S*, 4*S*, 5*R*, 7*S*.

The biosynthetic relationship of all isolated compounds (**1**–**6**) biosynthesis of compounds **1**–**6** was proposed as shown in Figure S54. Briefly, the eremophilane sesquiterpene core (I) and acorenyl cation (XI), both derived from farnesyl pyrophosphate (FPP), were considered as substrates to produce compounds **1**–**6** through a series of biochemical transformations, such as hydroxylation, isomerization, epoxidation, and acetylation [17,20].

### 2.2. COX-2 Inhibitory Activity

**Figure 6:**
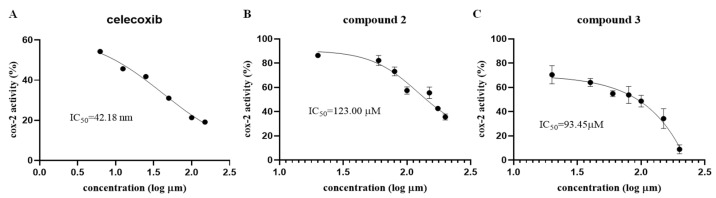
The inhibitory effects (IC_50_) of celecoxib and compounds **2** and **3** toward COX-2. (**A**) Celecoxib (positive control): 42.18 nM. (**B**) Compound **2**: 123.00 μM. (**C**) Compound **3**: 93.45 μM.

Cyclooxygenases 2 (COX-2) is involved in the key step of the converting arachidonic acid into prostaglandins, which are considered as therapeutic targets for many inflammatory related diseases [21,22]. Compounds with similar structures as described in the present work showed anti-inflammatory activities [9,19], so all of the isolated compounds (**1**–**6**) were screened for their anti-inflammatory activity at 100 µM towards COX-2 in vitro, among which, the eremophilane derivatives **2** and **3** were further assayed with concentration gradients and showed slight inhibitory effects with IC_50_ values 123.00 and 93.45 µM, respectively (Figure 6), whereas compounds **1** and **4**–**6** did not show any activities during the assays. Moreover, none of the isolated compounds showed any antimicrobial activities against agricultural pathogenic fungal strains *Ceratobasidium cornigerum* QDAU-6, *Botrytis cinerea* QDAU-23, *Fusarium oxysporum* QDAU-5, and aquatic pathogenic bacterial strains *Escherichia coli* EMBLC-1, *Vibrio anguillarum* QDIO-6, *Vibrio harveyi* QDIO-7. As well, the inhibitory activities against neuraminidase and angiotensin I converting enzyme 2 (ACE 2) were also tested and no obvious activities were observed.

**Figure 7:**
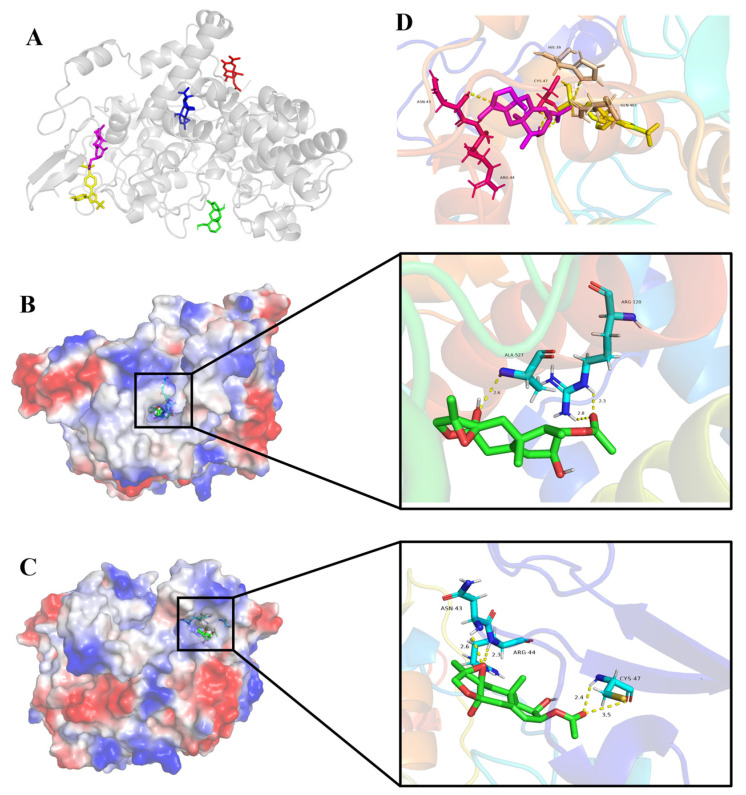
Molecular docking simulation results: (**A**) Compounds **1**–**4** and celecoxib interacted with 5IKR (**1** in red, **2** in blue, **3** in pink, **4** in green, celecoxib in yellow); (**B**) Compound **2** interacted with 5IKR; (**C**) Compound **3** interacted with 5IKR; (**D**) Compound **3** and celecoxib interacted with 5IKR.

A molecular docking study was performed to identify the intermolecular interaction and potential binding sites of the eremophilane derivatives **1**–**4** and the positive control celecoxib with COX-2 (PDB ID:5IKR) (Figure 7A). The results demonstrated that compound **3** interacted with COX-2 in the same active pocket with that of celecoxib (Figure 7D), which formed four key hydrogen bonds with residues Asn-43, Arg-44, and Cys-47, while celecoxib formed hydrogen bonds with residues His-39 and Gln-461 (Figure 7C,D). Hence, the huge variation of the inhibition activities might be implicated by their different nature of interactions with different residues of COX-2. In contrast, compound **2** was observed to form three hydrogen bonds with residues Arg-120 and Ala-527 (Figure 7B). The different locations of acetoxyl group in **2** and **3** exhibited great influence on the binding sites.

## 3. Material and Methods

### 3.1. General Experimental Procedures

Detailed information for the apparatus, reagents, solvents, and materials used in the present work is the same as that described in our previous publication [23,24,25]. Optical rotations were measured in MeOH using an Optical Activity AA-55 polarimeter (Optical Activity Ltd., Cambridgeshire, UK). UV spectra were measured on a PuXi TU-1810 UV-visible spectrophotometer (Shanghai Lengguang Technology Co., Ltd., Shanghai, China). ECD spectra were acquired on a Chirascan spectropolarimeter (Applied Photophysics Ltd., Surrey, UK). 1D and 2D NMR spectra were recorded at 500 and 125 MHz for ^1^H and ^13^C, respectively, on a Bruker Avance 500 spectrometer with TMS as internal standard (Bruker Biospin Group, Karlsruhe, Germany). Mass spectra were determined on an API QSTAR Pulsar 1 mass spectrometer (Applied Biosystems, Foster City, CA, USA). Analytical and semi-preparative HPLC were performed using a Dionex HPLC system (Dionex, Sunnyvale, CA, USA) equipped with P680 pump, an ASI-100 automated sample injector, and a UVD340U multiple wavelength detector controlled by Chromeleon software (version 6.80). Commercially available Si gel (200–300 mesh, Qingdao Haiyang Chemical Co., Qingdao, China), Lobar LiChroprep RP–18 (40–63 μm, Merck, Darmstadt, Germany), and Sephadex LH–20 (Merck, Darmstadt, Germany) were used for open column chromatography. All solvents used were distilled prior to use.

### 3.2. Fungal Material

The fungal strain *Furcasterigmium furcatum* CS-280 was isolated from sediment samples collected from the cold-seep environment of the South China Sea (depth 1183 m), in June 2020. The fungal strain was identified as *Furcasterigmium furcatum* (syn. *Acremonium furcatum*) according to its ITS region sequence, which is highly similar (98.93%) to that of *Acremonium furcatum* (LR026839.1). The sequence information of the fungus was deposited in GenBank with the accession no. OQ120564.1. The strain is preserved at the Key Laboratory of Experimental Marine Biology, Institute of Oceanology, Chinese Academy of Sciences (IOCAS). Detailed information for the isolation and identification of fungal strain *F. furcatum* CS-280 was described in the previous publication [10].

### 3.3. Fermentation, Extraction, and Isolation

For chemical investigations, the bacterial strain *P. aeruginosa* QDIO-4 was incubated in LB liquid medium (10 g tryptone, 5 g yeast extract, and 10 g NaCl in 1 L distilled H_2_O, pH 7.0) at 37 °C, 200 rpm for 12 h, and then 10 mL of bacterial broth was inoculated into 200 × 1 L conical flasks with solid rice medium (100 g rice, 0.15 g corn syrup, 0.45 g peptone, 0.15 g methionine, and 150 mL naturally sourced and filtered seawater that was obtained from the Huiquan Gulf of the Yellow Sea near the campus of IOCAS) at room temperature. After 3 days, these flasks were autoclaved at 121 °C for 20 min, and then a piece of the spore inoculum (~2 × 2 cm^2^) of the fungal strain *F. furcatum* CS-280, which was grown on PDA medium at 28 °C for 8 days, was introduced into the above mentioned autoclaved rice-substrate-containing flasks and further incubated for 44 days. The fermented rice substrate was extracted four times with EtOAc.

The EtOAc extract was filtered and evaporated under reduced pressure to yield an organic extract (80.2 g), which was subjected to vacuum liquid chromatography (VLC) over Si gel eluting with different solvents of increasing polarity from petroleum ether (PE) to MeOH to yield nine fractions (Frs. 1–9).

Based on TLC and HPLC analysis, Fr. 4 (9.8 g) and Fr. 5 (6.2 g) were combined together for further isolation, yielding 9 subfractions (Frs. 4.1–4.9) by column chromatography (CC) over Lobar LiChroprep RP-18 with a MeOH–H_2_O gradient (from 10:90 to 90:10). Fr 4.3 was applied to CC on Si gel eluting with a PE–acetone gradient (from 15:1 to 5:1) and then purified by CC on Sephadex LH-20 (MeOH) to yield compound **6** (2.0 mg). Fr. 6 (2.0 g) and Fr. 7 (16.0 g) were mixed together and then yielded nine subfractions (Frs. 6.1–6.9) by CC over Lobar LiChroprep RP-18 with a MeOH–H_2_O gradient (from 10:90 to 90:10). Fr. 6.2 was applied to CC on Si gel eluting with a PE–acetone gradient (from 5:1 to 1:1) and then purified by CC on Sephadex LH-20 (MeOH) to yield compound **4** (5.0 mg). Fr. 6.3 was purified by semipreparative HPLC (Elite ODS-BP column, 10 μm; 20 × 250 mm; 41% MeOH–H_2_O, 12 mL/min) to obtain compounds **2** (2.3 mg) and **5** (2.1 mg), while the eluents of un-purified peaks were collected and dried in vacuum to get the remaining components, and were further purified by CC on Si gel eluting with a gradient CH_2_Cl_2_–MeOH, 100:1 to obtain compound **3** (3.0 mg). Fr 6.6 and 6.7 was applied to semipreparative HPLC (Elite ODS-BP column, 10 μm; 20 × 250 mm; 66% MeOH–H_2_O, 12 mL/min) and then was further purified by CC on Sephadex LH-20 (MeOH) to yield compound **1** (2.0 mg).

Fureremophilane A (**1**): colorless crystals (MeOH–H_2_O); ${\left[\alpha \right]}_{D}^{25}$ + 35.3 (*c* 0.17, MeOH); UV (MeOH) λ_max_ (log ε) 200 (3.21), 220 (2.69) 245 (2.98) 280 (2.76) nm; ECD (0.68 mM, MeOH) λ_max_ (Δε) 205 (+4.75), 242 (+12.43), 260 (+3.94), 281 (−4.49) nm; ^1^H and ^13^C NMR data, see Table 1 and Table 2; HRESIMS m/z 293.1751 [M + H]^+^ (C_17_H_25_O_4_, calcd 293.1747).

Fureremophilane B (**2**): yellow oil; ${\left[\alpha \right]}_{D}^{25}$ + 31.3 (*c* 0.16, MeOH); UV (MeOH) λ_max_ (log ε) 200 (3.12), 220 (2.49) 242 (2.43) 320 (2.11) nm; ECD (0.62 mM, MeOH) λ_max_ (Δε) 200 (+8.38), 215 (−0.99), 240 (+0.74) nm; ^1^H and ^13^C NMR data, see Table 1 and Table 2; HRESIMS *m*/*z* 347.1469 [M + Na]^+^ (C_17_H_24_O_6_Na, calcd 347.1465); 325.1648 [M + H]^+^ (C_17_H_25_O_6_, calcd 325.1646).

Fureremophilane C (**3**): colorless crystals (MeOH–EtOAc); ${\left[\alpha \right]}_{D}^{25}\;$ + 43.8 (*c* 0.16, MeOH); UV (MeOH) λ_max_ (log ε) 200 (3.11), 220 (2.45) 283 (2.19) nm; ECD (0.62 mM, MeOH) λ_max_ (Δε) 200 (+7.96) nm; ^1^H and ^13^C NMR data, see Table 1 and Table 2; HRESIMS *m*/*z* 347.1462 [M + Na]^+^ (C_17_H_24_O_6_Na, calcd 347.1465).

Fureremophilane D (**4**): colorless crystals (MeOH–H_2_O); ${\left[\alpha \right]}_{D}^{25}$ + 36.0 (*c* 0.25, MeOH); UV (MeOH) λ_max_ (log ε) 203 (+3.94) nm; ^1^H and ^13^C NMR data, see Table 1 and Table 2; HRESIMS *m*/*z* 289.1404 [M + Na]^+^ (C_15_H_22_O_4_Na, calcd 289.1410).

Furacorane A (**5**): yellowish oil; ${\left[\alpha \right]}_{D}^{25}$ − 18.2 (*c* 0.11, MeOH); UV (MeOH) λ_max_ (log ε) 200 (3.05) nm; ^1^H and ^13^C NMR data, see Table 3; HRESIMS *m*/*z* 255.1958 [M + H]^+^ (C_15_H_27_O_3_, calcd 255.1955).

Furacorane B (**6**): yellowish oil; ${\left[\alpha \right]}_{D}^{25}$ +76.5 (*c* 0.17, MeOH); UV (MeOH) λ_max_ (log ε) 200 (2.55) nm; ECD (4.24 mM, MeOH) λ_max_ (Δε) 295 (+1.51) nm; H and ^13^C NMR data, see Table 3; HRESIMS *m*/*z* 254.2121 [M + NH_4_]^+^ (C_15_H_28_O_2_N, calcd 254.2115).

### 3.4. X-Ray Crystallographic Analysis of Compounds **1**, **3** and **4** [26]

Crystallographic data were collected on a Srigaku Mercury CCD/AFCR diffractometer equipped with graphite-monochromatic Cu K*α* radiation (λ = 1.541 78 Å) at 293(2) K. The data were corrected for absorption by using the program SADABS [27]. The structure was solved by direct methods and subsequent difference Fourier synthesis and refined by full-matrix least-squares techniques with the SHELXTL software package (2018) [28,29]. All non-hydrogen atoms were refined anisotropically. The absolute structures were determined by refinement of the Flack parameter [30].

*Crystal data of* **1**: m.p. 167–169 ℃; C_17_H_24_O_4_; F.W. = 292.36, Orthorhombic, *P2_1_2_1_2_1_*, unit cell dimensions *a* = 7.3305(2) Å, *b* = 9.8107(3) Å, *c* = 21.7397(6) Å, *V* = 1563.46(8) Å^3^, *α* = *β* = *γ* = 90°, *Z* = 4, d_calcd_ =1.242 mg/m^3^, crystal dimensions 0.160 × 0.140 × 0.120 mm, *μ* = 0.706 mm^−1^, *F*(000) = 632.0. The 15680 measurements yielded 2848 independent reflections after equivalent data were averaged, and Lorentz and polarization corrections were applied. The final refinement gave *R*_1_ = 0.0307 and w*R*_2_ = 0.0853 [*I* > 2*σ*(*I*)]. The Flack parameter was 0.09(5).

*Crystal data of* **3**: m.p. 170–172 ℃; C_17_H_24_O_6_; F.W. = 324.36, Trigonal, *R3*, unit cell dimensions *a* = 27.6010(6) Å, *b* = 27.6010(6) Å, *c* = 6.1835(2) Å, *V* = 4079.6(2) Å^3^, *α* = *β* = 90°, *γ* =120°, *Z* = 9, d_calcd_ =1.188 mg/m^3^, crystal dimensions 0.140 × 0.120 × 0.100 mm, *μ* = 0.743 mm^−1^, *F*(000) = 1566.0. The 23292 measurements yielded 3332 independent reflections after equivalent data were averaged, and Lorentz and polarization corrections were applied. The final refinement gave *R*_1_ = 0.0307 and w*R*_2_ = 0.0848 [*I* > 2*σ*(*I*)]. The Flack parameter was 0.08(4).

*Crystal data of* **4**: m.p. 208–210 °C; C_15_H_22_O_4_; F.W. = 266.32, Orthorhombic, *P2_1_2_1_2_1_*, unit cell dimensions *a* = 9.6756(2) Å, *b* = 10.8136(2) Å, *c* = 12.8771(3) Å, *V* = 1347.31(5) Å^3^, *α* = *β* = *γ* = 90°, *Z* = 4, d_calcd_ = 1.313 mg/m^3^, crystal dimensions 0.180 × 0.160 × 0.120 mm, *μ* = 0.766 mm^−1^, *F*(000) = 576.0. The 6498 measurements yielded 2404 independent reflections after equivalent data were averaged, and Lorentz and polarization corrections were applied. The final refinement gave *R*_1_ = 0.0309 and w*R*_2_ = 0.0843 [*I* > 2*σ*(*I*)]. The Flack parameter was 0.13(8).

### 3.5. Specific Rotation and ECD Calculations

Conformational searches were carried out via molecular mechanics with the MM+ method in HyperChem 8.0 software. Afterward, the geometries were optimized at the gas-phase B3LYP/6-31G(d) level in Gaussian09 software to afford the energy-minimized conformers [31]. Then, the optimized conformers were subjected to the calculations of ECD spectra and specific rotation value (λ = 589.4 nm) using the CAM-B3LYP/TZVP level. Solvent effects of the methanol solution were evaluated at the same DFT levels using the SCRF/PCM method. The calculated results were later obtained according to the Boltzmann weighting of each conformer.

### 3.6. COX-2 Inhibitory Assays

COX-2 inhibitory activity assay in vitro was performed by cyclooxygenase-2 Inhibitor Screening Kit (Beyotime, Cat No. S0168, Shanghai, China). Briefly, the COX-2 enzyme was mixed with compound solution and then incluated at 37 °C for 10 min. After that, the COX-2 probe and substrate were added. Then, the fluorescence intensity values were measured with the excitation wavelength at 530 nm and the emission wavelength at 590 nm. The data was analyzed by GraphPad Prism5 (GraphPad Software Inc., Boston, MA, USA).

### 3.7. Molecular Docking Simulations

The crystal structure of human COX-2 (PDB ID: 5IKR) was downloaded from the Protein Data Bank for molecular docking. The preparation, optimization, and interaction of compounds **1**–**4** and celecoxib bonding with COX-2 and legitimacy of their activities were elaborated by AutoDock software package (http://autodock.scripps.edu/, accessed on 15 October 2024, Molecular Graphics Laboratory at the Department of Molecular Biology, The Scripps Research Institute). AutoDock tools were employed to prepare both COX-2 and compounds for docking. Through AutoDock vina, binding free energy was calculated and compounds displaying the lowest binding affinity to protein were chosen as the best conformation [32]. The visualizations of the protein-ligand structure were optimized by PyMol molecular graphics system.

## 4. Conclusions

Six new sesquiterpenes, including four eremophilane derivatives, fureremophilanes A–D (**1**–**4**), and two acoranes, furacoranes A and B (**5** and **6**), were characterized from the culture extract of the cold-seep derived fungus *Furcasterigmium furcatum* CS-280 cultured on autoclaved *P. aeruginosa* QDIO-4. The compounds with more variable structures were isolated from the extract of co-culture medium, while at the same time, the identified compounds were highly oxidized and compounds **2** and **3** had rare epoxyethane and tetrahydrofuran ring systems among the known sesquiterpenes. Compounds **2** and **3** displayed inhibitory activities against human COX-2 activity with IC_50_ values 123.00 and 93.45 µM, respectively. The molecular docking experiment was carried out to explain their distinct activities. Compound **3** was considered to be engaged in the same active pocket with celecoxib, while the different binding residues lead to the difference in the inhibitory activities of COX-2 in vitro, and the different locations of acetyl group in **2** and **3** exhibited great influence on the binding sites.

## Figures and Tables

**Table 1:** ^1^H NMR Data of Compounds **1**–**4** in DMSO-*d*_6_ (*δ* in ppm, *J* in Hz).

no.	1	2	3	4
1	2.55, dd (13.1, 2.6) 2.30, dd (13.1, 5.6)	5.23, s	5.12, d (2.4)	2.00, td (13.4, 4.2) 0.96, overlap
2	3.63, m	4.19, t (6.0)	5.14, dd (4.4, 2.4)	1.67, d (11.1) 1.38, m
3	5.09, t (2.9)	5.06, d (6.0)	3.78, td (4.4, 2.2)	1.82, d (10.6) 1.28, overlap
4	1.74, qd (7.0, 2.9)	1.76, q (7.2)	1.58, overlap	1.33, overlap
6	2.86, d (13.9) 1.98, overlap	1.80, d (13.2) 1.56, d (13.2)	1.80, d (13.2) 1.55, d (13.2)	1.61, dd (13.3, 6.0) 1.14, t (13.3)
7	-	-	-	2.39, dd (13.3, 6.0)
9	5.74, s	2.54, d (13.1) 2.27, d (13.1)	2.52, overlap 2.28, d (13.0)	2.92, s
12	2.00, s	3.65, d (9.6) 3.61, d (9.6)	3.64, d (9.7) 3.61, d (9.7)	4.34, d (12.9) 4.29, overlap
13	1.81, s	1.36, s	1.36, s	4.90, br s
14	1.02, s	1.13, s	1.18, s	0.99, s
15	0.92, d (7.0)	0.90, d (7.2)	1.03, d (7.0)	3.57, d (10.4) 3.11, d (10.4)
17	2.07, s	1.99, s	2.03, s	
2-OH	5.05, d (6.0)	4.75, d (6.0)		
3-OH			4.64, d (4.4)	
8-OH		5.82, s	5.80, s	6.18, s
15-OH				4.30, overlap

**Table 2:** ^13^C NMR data of compounds **1**–**4** in DMSO-*d*_6_ (*δ* in ppm).

no.	1	2	3	4
1	35.8, CH_2_	126.5, CH	121.1, CH	29.3, CH_2_
2	68.5, CH	66.0, CH	72.2, CH	22.6, CH_2_
3	75.2, CH	73.8, CH	69.0, CH	24.8, CH_2_
4	41.4, CH	40.8, CH	41.6, CH	46.9, CH
5	39.9, C	38.6, C	39.5, C *^a^*	35.6, C
6	40.7, CH_2_	35.2, CH_2_	35.3, CH_2_	35.7, CH_2_
7	127.3, C	67.9, C	67.9, C	44.4, CH
8	190.2, C	100.7, C	100.7, C	101.7, C
9	126.9, CH	39.6, CH_2_	39.5, CH_2_	62.1, CH
10	165.3, C	139.7, C	142.6, C	64.8, C
11	142.4, C	61.6, C	61.4, C	152.1, C
12	22.1, CH_3_	68.6, CH_2_	68.6, CH_2_	68.3, CH_2_
13	21.7, CH_3_	11.2, CH_3_	11.2, CH_3_	103.8, CH_2_
14	17.9, CH_3_	19.4, CH_3_	19.7, CH_3_	16.0, CH_3_
15	11.3, CH_3_	12.6, CH_3_	12.8, CH_3_	61.4, CH_2_
16	170.0, C	170.2, C	170.1, C	
17	20.8, CH_3_	21.0, CH_3_	21.0, CH_3_	

*^a^* Observed in the HMBC spectrum.

**Table 3:** ^1^H and ^13^C NMR data of compounds **5** and **6** in DMSO-*d*_6_ (*δ* in ppm, *J* in Hz).

no.	5		no.	6	
	*δ*_H_ ^a^ (mult, *J* in Hz)	*δ*_C_ ^b^, Type		*δ*_H_ ^a^ (mult, *J* in Hz)	*δ*_C_ ^b^, Type
1	1.43, dt (10.7, 8.3)	56.9, CH	1	1.89, overlap	45.1, CH
2	1.78, overlap 1.51, overlap	37.0, CH_2_	2	2.26, dd (19.5, 9.2) 2.12, dd (19.5, 4,7)	36.6, CH_2_
3	3.53, td (9.0, 4.9)	74.3, CH	3		220.5, C ^c^
4	1.36, m	55.1, CH	4	2.50, m	50.4, CH
5		44.8, C	5		44.4, C
6	1.55, overlap 1.20, dd (13.4, 9.9)	32.9, CH_2_	6	1.94, overlap 1.61, overlap	37.6, CH_2_
7	4.18, m	64.5, CH	7	4.10, m	66.2, CH
8		140.8, C	8		136.4, C
9	5.56, s	121.4, CH	9	5.32, m	120.8, CH
10	2.06, m 1.83, overlap	34.9, CH_2_	10	1.87, overlap 1.63, overlap	30.7, CH_2_
11	1.59, overlap	29.4, CH	11	2.03, m	26.9, CH
12	0.86, d (6.5)	23.3, CH_3_	12	0.87, d (6.9)	23.7, CH_3_
13	0.80, d (6.5)	22.6, CH_3_	13	0.82, d (6.8)	18.9, CH_3_
14	0.83, d (6.9)	11.7, CH_3_	14	0.80, d (7.2)	9.7, CH_3_
15	3.97, br s	61.5, CH_2_	15	1.68, s	19.7, CH_3_
3-OH	4.47, d (4.9)		7-OH	4.77, d (5.8)	
7-OH	4.53, d (6.1)				
15-OH	4.42, t (5.5)				

^a^ Measured at 500 MHz, ^b^ Measured at 125 MHz, ^c^ Observed in the HMBC spectrum.

## Data Availability

The data are included in the article and the Supplementary Material.

## Supplementary Materials

The following supporting information can be downloaded at: https://www.mdpi.com/article/10.3390/md22120574/s1. Tables S1–S3: Calculated specific rotation values. Figures S1–S3: Chemical structures, optimized low-energy conformers for ECD and SR calculations. Figures S4–S52: The HRESIMS, 1D and 2D NMR spectra, ECDs of compounds **1**–**6**. Figure S53: Chart flow of the isolation procedure. Figure S54: Proposed biogenetic network for compounds 1–6.
