# Supplementary material for: Eremophilane- and Acorane-Type Sesquiterpenes from the Deep-Sea Cold-Seep-Derived Fungus Furcasterigmium furcatum CS-280 Cultured in the Presence of Autoclaved Pseudomonas aeruginosa QDIO-4
Source: Mar Drugs. 2024 Dec 22;22(12):574. doi: 10.3390/md22120574 (PMC11677535; doi:10.3390/md22120574)

Supplementary data

Eremophilane- and Acorane-Type Sesquiterpenes from the Deep-Sea Cold-Seep-Derived Fungus Furcasterigmium furcatum CS-280 Cultured in the Presence of Autoclaved Pseudomonas aeruginosa QDIO-4

Xiao-Dan Chen 1,2, Xin Li 1,2,3, Xiao-Ming Li 1,3, Sui-Qun Yang 1,2,3,* and Bin-Gui Wang 1,2,3,*

[1] *CAS and Shandong Province Key Laboratory of Experimental Marine Biology, Institute of Oceanology, Chinese Academy of Sciences, Nanhai Road 7, Qingdao 266071, China*

[2] *University of Chinese Academy of Sciences, Yuquan Road 19A, Beijing 100049, China*

[3] *Laboratory for Marine Biology and Biotechnology, Qingdao Marine Science and Technology Center, Wenhai Road 1, Qingdao 266237, China*

[*] Correspondence: yangsuiqun@qdio.ac.cn (S.-Q.Y.); wangbg@ms.qdio.ac.cn (B.-G.W.); Tel.: +86-532-82898890 (S.-Q.Y.); +86-532-82898553 (B.-G.W.)

Content

Table S1. Calculated specific rotation values at 589.44 nm for the enantiomers (1*S*, 3*S*, 4*S*, 5*R*, 7*S*)-**5** and (1*R*, 3*R*, 4*R*, 5*S*, 7*R*)-**5**;

Table S2. Calculated specific rotation values for conformers A−G of (1*S*, 3*S*, 4*S*, 5*R*, 7*S*)-**5**;

Table S3. Calculated specific rotation values for conformers A−J of (1*R*, 3*R*, 4*R*, 5*S*, 7*R*)-**5**;

Figure S1. Optimized geometries of predominant conformers (weighting factors) for (1*S*, 3*S*, 4*S*, 5*R*, 7*S*)-**5** at the B3LYP/6-31g(d) level;

Figure S2. Optimized geometries of predominant conformers (weighting factors) for (1*R*, 3*R*, 4*R*, 5*S*, 7*R*)-**5** at the B3LYP/6-31g(d) level;

Figure S3. Optimized geometries of predominant conformers (weighting factors) for (1*S*, 4*S*, 5*R*, 7*S*)-**6** at the B3LYP/6-31g(d) level;

Figure S4. ^{1}H NMR (500 MHz, DMSO-d_6) spectrum of compound **1**;

Figure S5. ^{13}C NMR (125 MHz, DMSO-d_6) and DEPT spectra of compound **1**;

Figure S6. COSY spectrum of compound **1**;

Figure S7. HSQC spectrum of compound **1**;

Figure S8. HMBC spectrum of compound **1**;

Figure S9. NOESY spectrum of compound **1**;

Figure S10. HRESI mass spectrum of compound **1**;

Figure S11. ECD spectrum of compound **1**;

Figure S12. ^{1}H NMR (500 MHz, DMSO-d_6) spectrum of compound **2**;

Figure S13. ^{13}C NMR (125 MHz, DMSO-d_6) and DEPT spectra of compound **2**;

Figure S14. COSY spectrum of compound **2**;

Figure S15. HSQC spectrum of compound **2**;

Figure S16. HMBC spectrum of compound **2**;

Figure S17. NOESY spectrum of compound **2**;

Figure S18. HRESI mass spectrum of compound **2**;

Figure S19. ECD spectrum of compound **2**;

Figure S20. ^{1}H NMR (500 MHz, DMSO-d_6) spectrum of compound **3**;

Figure S21.^{13}C NMR (125 MHz, DMSO-d_6) and DEPT spectra of compound **3**;

Figure S22. COSY spectrum of compound **3**;

Figure S23. HSQC spectrum of compound **3**;

Figure S24. HMBC spectrum of compound **3**;

Figure S25. NOESY spectrum of compound **3**

Figure S26. HRESI mass spectrum of compound **3**;

Figure S27. ECD spectrum of compound **3**;

Figure S28. ^{1}H NMR (500 MHz, DMSO-d_6) spectrum of compound **4**;

Figure S29.^{13}C NMR (125 MHz, DMSO-d_6) and DEPT spectra of compound **4**;

Figure S30. COSY spectrum of compound **4**;

Figure S31. HSQC spectrum of compound **4**;

Figure S32. HMBC spectrum of compound **4**;

Figure S33. NOESY spectrum of compound **4;**

Figure S34. HRESI mass spectrum of compound **4**;

Figure S35. ^{1}H NMR (500 MHz, DMSO-d_6) spectrum of compound **5**;

Figure S36.^{13}C NMR (125 MHz, DMSO-d_6) and DEPT spectra of compound **5**;

Figure S37. COSY spectrum of compound **5**;

Figure S38. HSQC spectrum of compound **5**;

Figure S39. HMBC spectrum of compound **5**;

Figure S40. NOESY spectrum of compound **5;**

Figure S41. ^{1}H NMR (600 MHz, $CDCl_3$) spectrum of compound **5**;

Figure S42. HSQC spectrum of compound **5** in $CDCl_3$;

Figure S43. NOESY spectrum of compound **5** in $CDCl_3$;

Figure S44. HRESI mass spectrum of compound **5**;

Figure S45. ^{1}H NMR (500 MHz, DMSO-d_6) spectrum of compound **6**;

Figure S46.^{13}C NMR (125 MHz, DMSO-d_6) and DEPT spectra of compound **6**;

Figure S47. COSY spectrum of compound **6**;

Figure S48. HSQC spectrum of compound **6**;

Figure S49. HMBC spectrum of compound **6**;

Figure S50. NOESY spectrum of compound **6**;

Figure S51. HRESI mass spectrum of compound **6**;

Figure S52. ECD spectrum of compound **6**;

Figure S53. Chart flow of the isolation procedure;

Figure S54. Proposed biogenetic network for compounds **1–6**.

Table S1. Calculated specific rotation values at 589.44 nm for the enantiomers (1*S*, 3*S*, 4*S*, 5*R*, 7*S*)-**5** and (1*R*, 3*R*, 4*R*, 5*S*, 7*R*)-**5**.

**specific rotation calculation**	**CAM-B3LYP/TZVP**
(1*S*, 3*S*, 4*S*, 5*R*, 7*S*)-**5**	-22.621571
(1*R*, 3*R*, 4*R*, 5*S*, 7*R*)-**5**	20.954645

Table S2. Calculated specific rotation values for conformers A−G of (1*S*, 3*S*, 4*S*, 5*R*, 7*S*)-**5**.

**conf.**	**CAM-B3LYP/TZVP**	**Boltzmann population**
A	-8.97	39.3%
B	22.38	16.6%
C	-29.12	16.5%
D	-29.93	10.7%
E	-139.78	7.1%
F	-55.36	3.1%
G	-106.73	3.0%

Table S3. Calculated specific rotation values for conformers A−J of (1*R*, 3*R*, 4*R*, 5*S*, 7*R*)-**5**.

**conf.**	**CAM-B3LYP/TZVP**	**Boltzmann population**
A	19.47	38.1%
B	-29.95	18.8%
C	-29.72	11.5%
D	23.62	8.8%
E	112.49	6.1%
F	87.56	3.9%
G	61.39	3.8%
H	103.99	3.4%
I	100.32	3.1%
J	49.59	2.4%

Figure S1. Optimized geometries of predominant conformers (weighting factors) for (1*S*, 3*S*, 4*S*, 5*R*, 7*S*)-**5** at the B3LYP/6-31g(d) level.

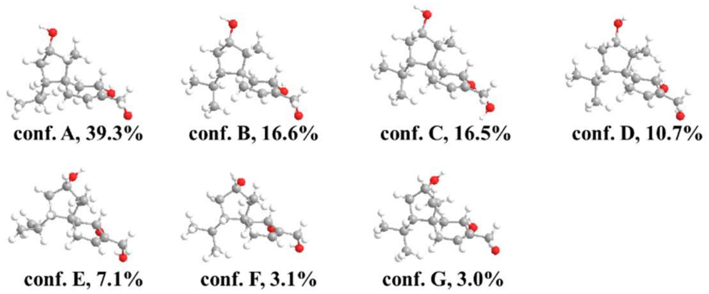

Figure S2. Optimized geometries of predominant conformers (weighting factors) for (1*R*, 3*R*, 4*R*, 5*S*, 7*R*)-**5** at the B3LYP/6-31g(d) level.

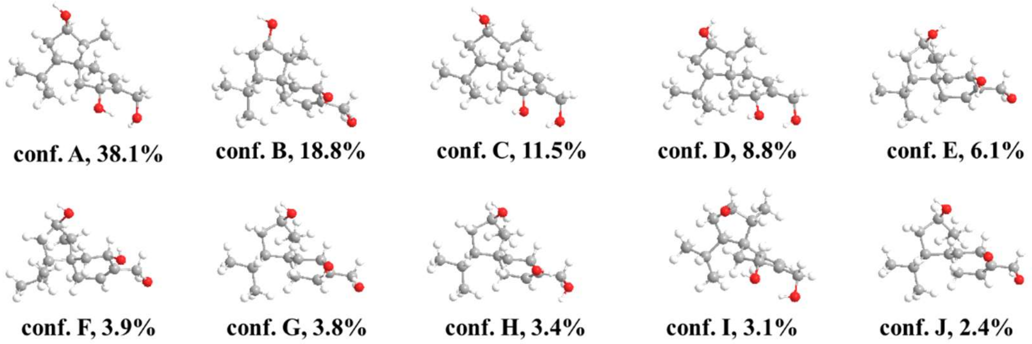

Figure S3. Optimized geometries of predominant conformers (weighting factors) for (1*S*, 4*S*, 5*R*, 7*S*)-**6** at the B3LYP/6-31g(d) level.

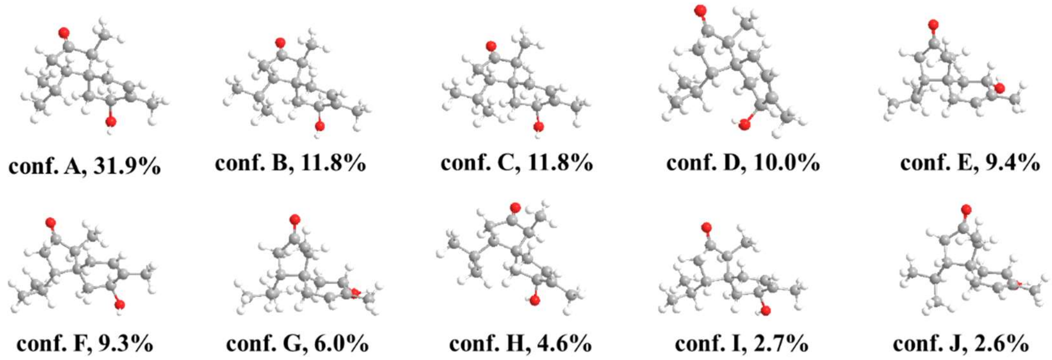

Figure S4. ^{1}H NMR (500 MHz, DMSO-d_6) spectrum of compound **1**.

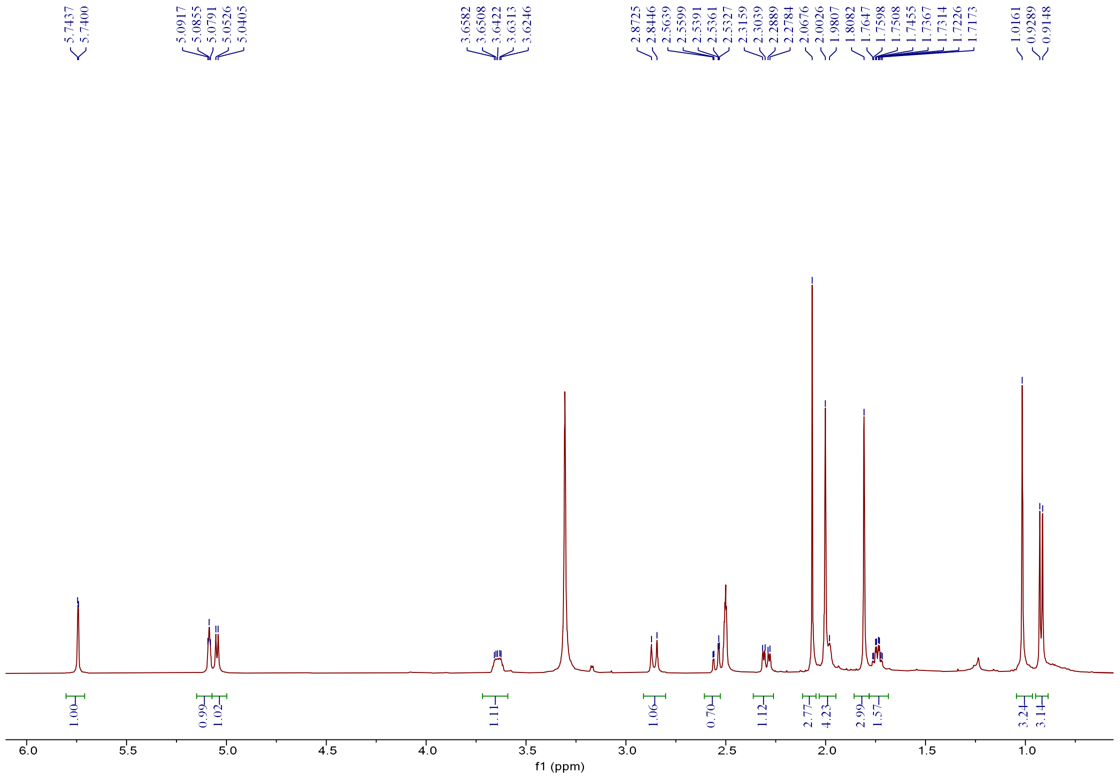

Figure S5. ^{13}C NMR (125 MHz, DMSO-d_6) and DEPT spectra of compound **1**.

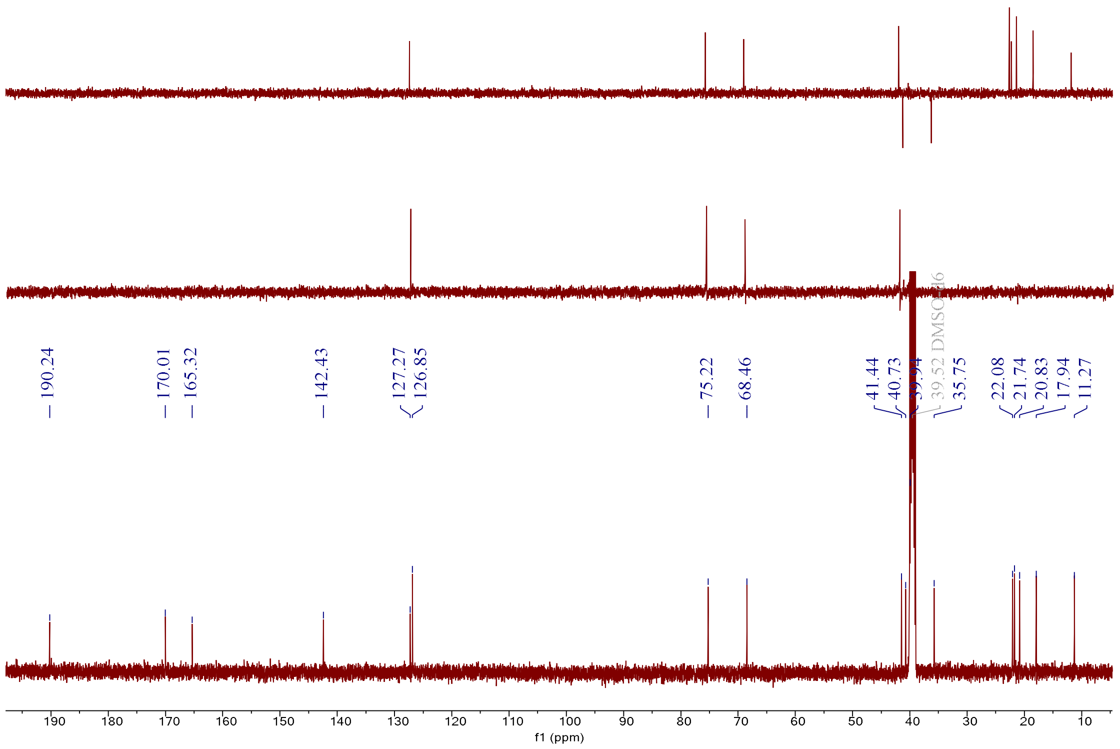

Figure S6. COSY spectrum of compound **1**.

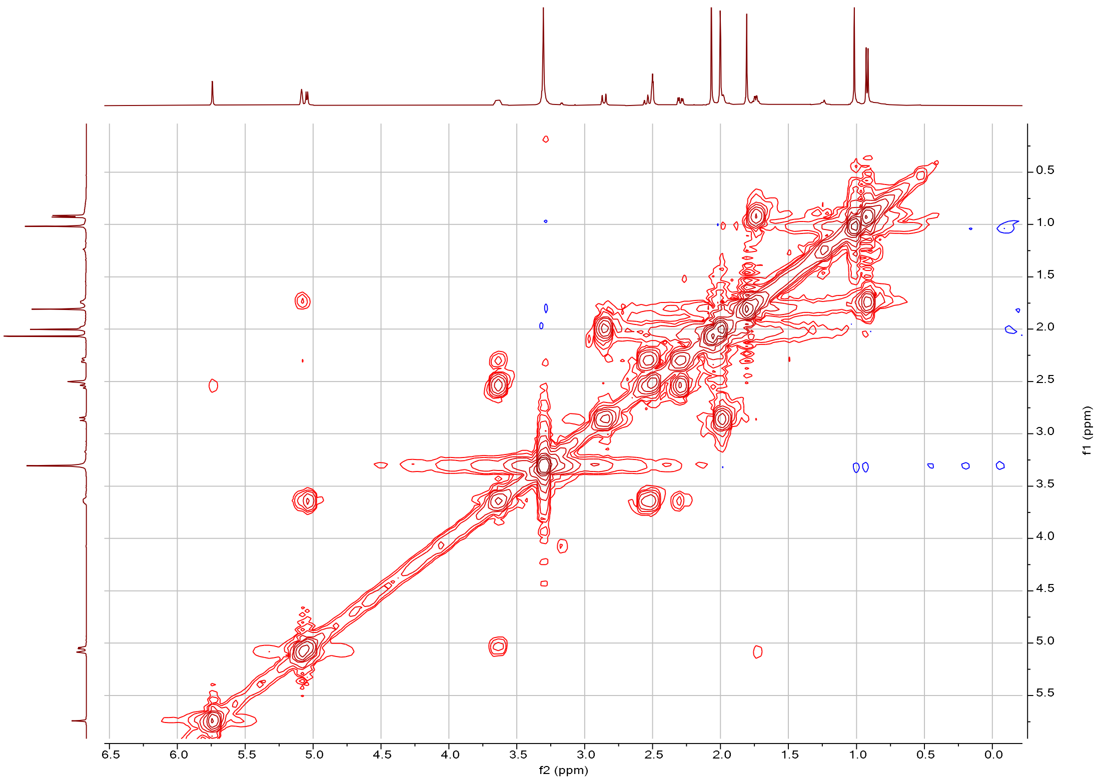

Figure S7. HSQC spectrum of compound **1**.

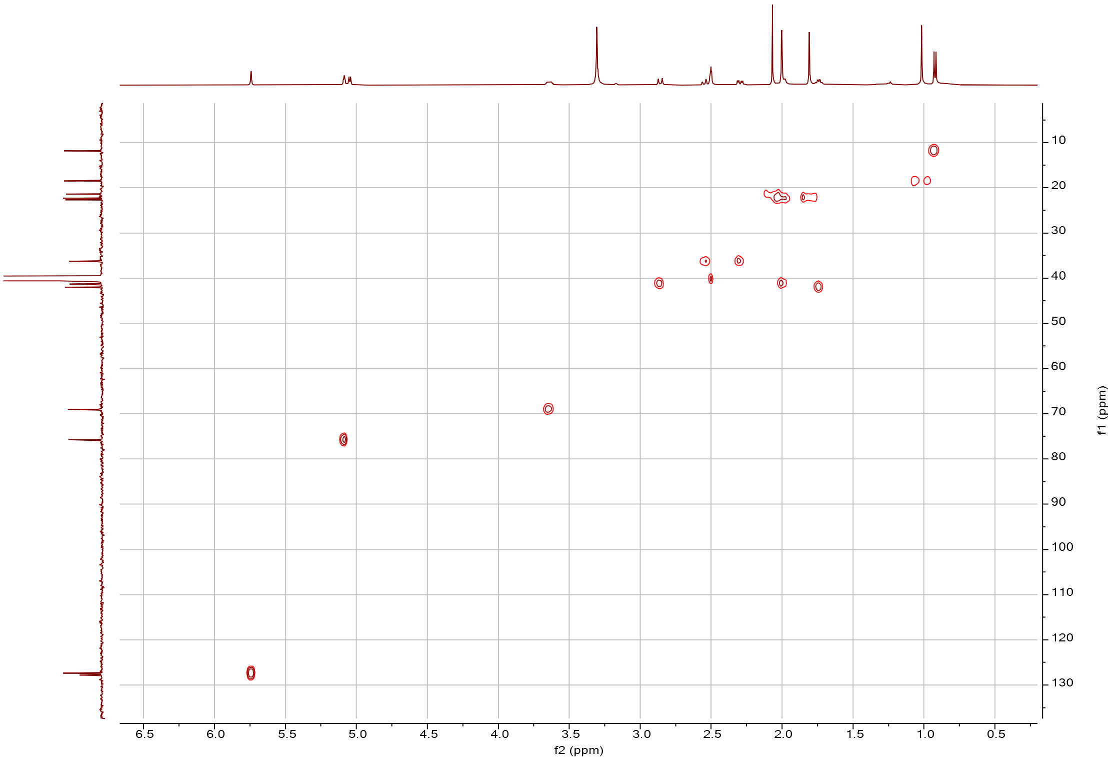

Figure S8. HMBC spectrum of compound **1**.

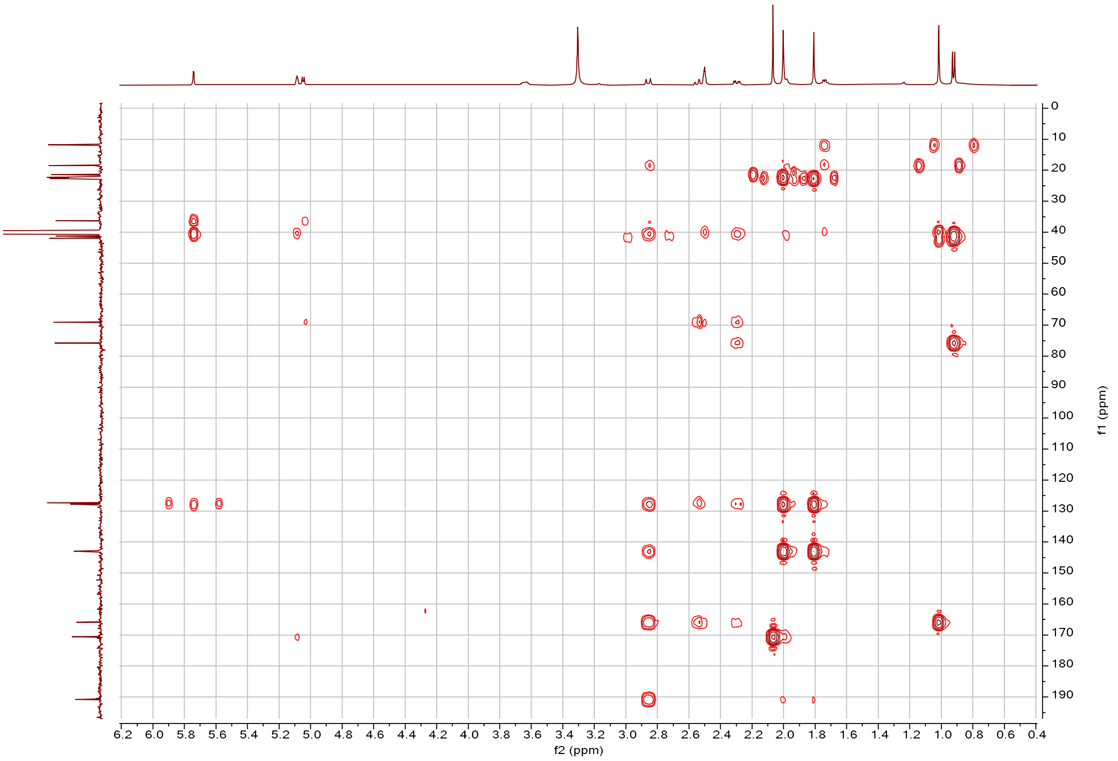

Figure S9. NOESY spectrum of compound **1**.

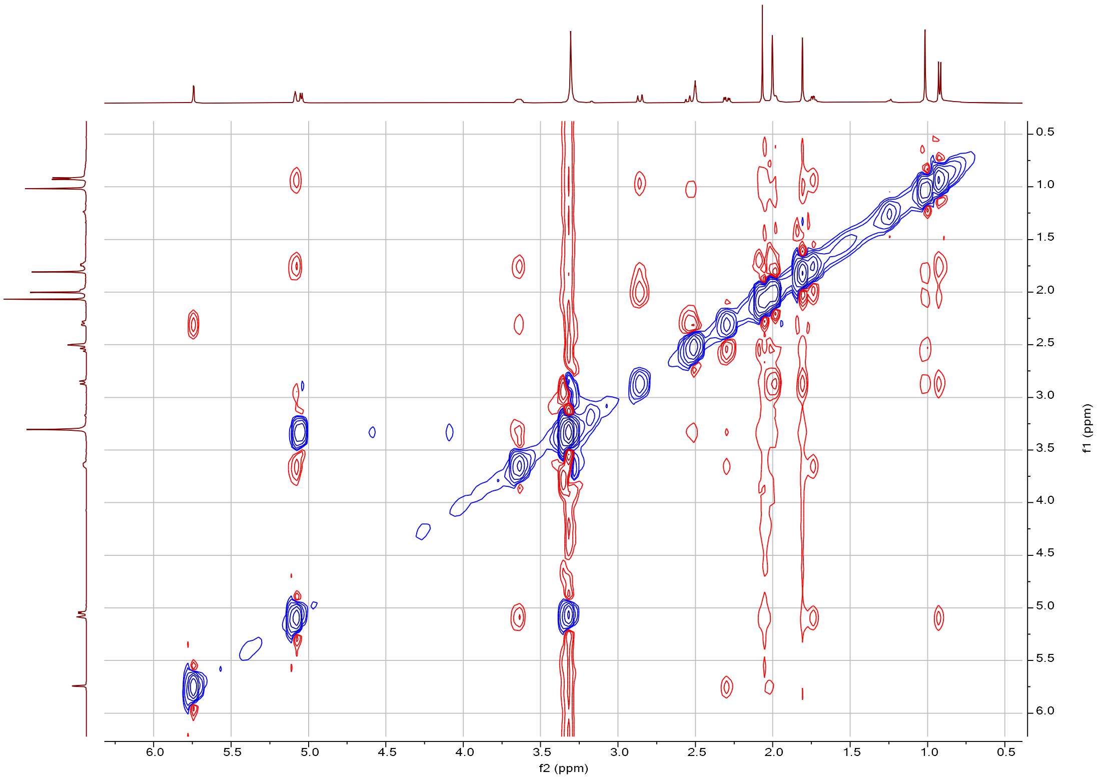

Figure S10. HRESI mass spectrum of compound **1**.

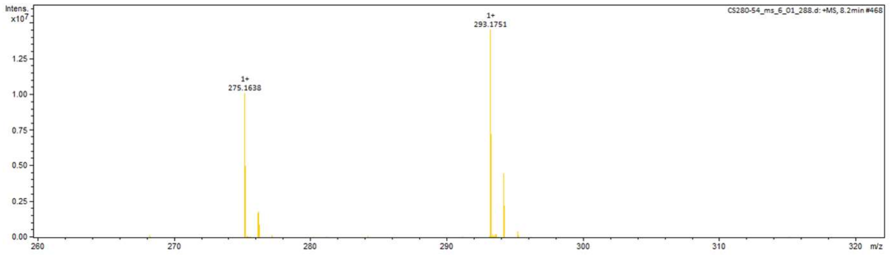

Meas. m/z	#	Ion Formula	Score	m/z	err [mDa]	err [ppm]	mSigma	rdb	e?Conf	N-Rule	Adduct	Sum Formula
293.175139	1	C17H25O4	100.00	293.174736	-0.4	-1.4	69.6	5.5	even	ok	M+H	C17H24O4

Figure S11. ECD spectrum of compound **1**.

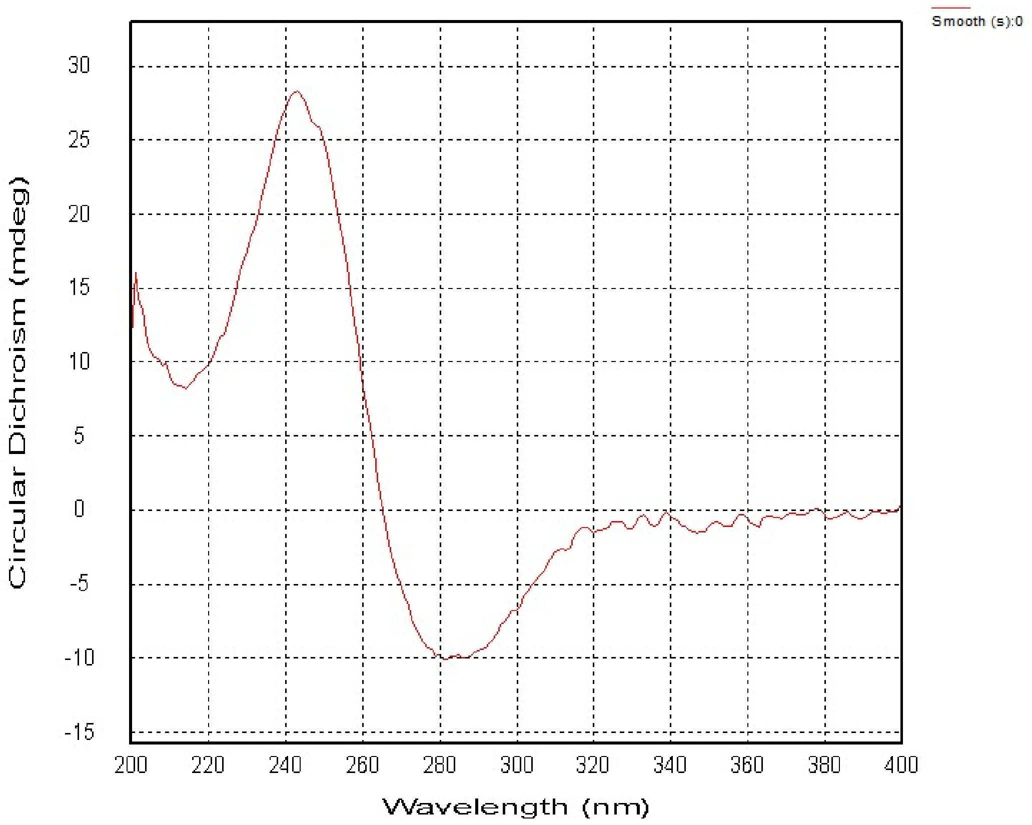

Figure S12. ^{1}H NMR (500 MHz, DMSO-d_6) spectrum of compound **2**.

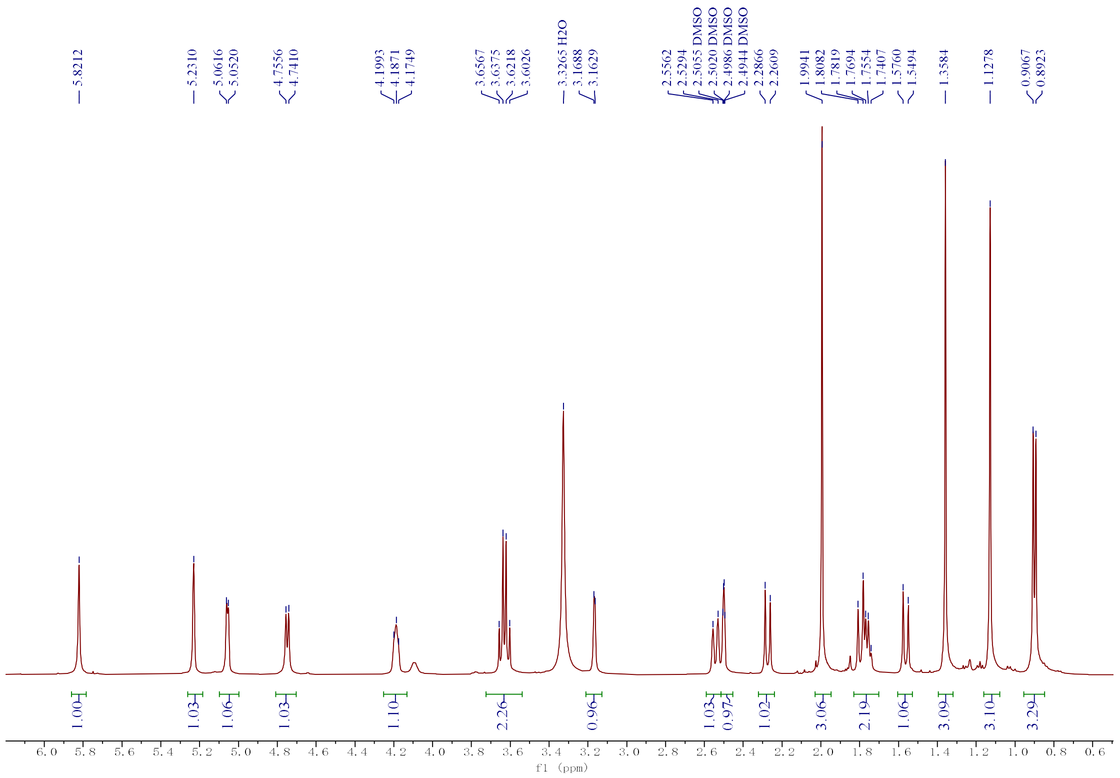

Figure S13. ^{13}C NMR (125 MHz, DMSO-d_6) and DEPT spectra of compound **2**.

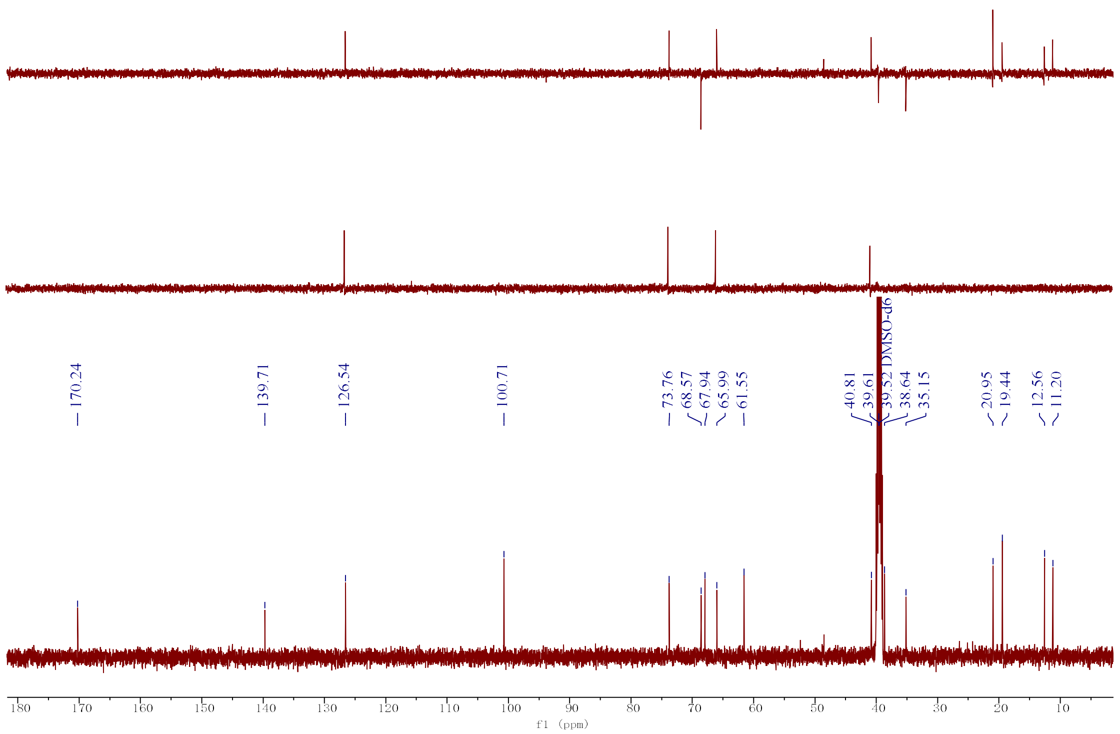

Figure S14. COSY spectrum of compound **2**.

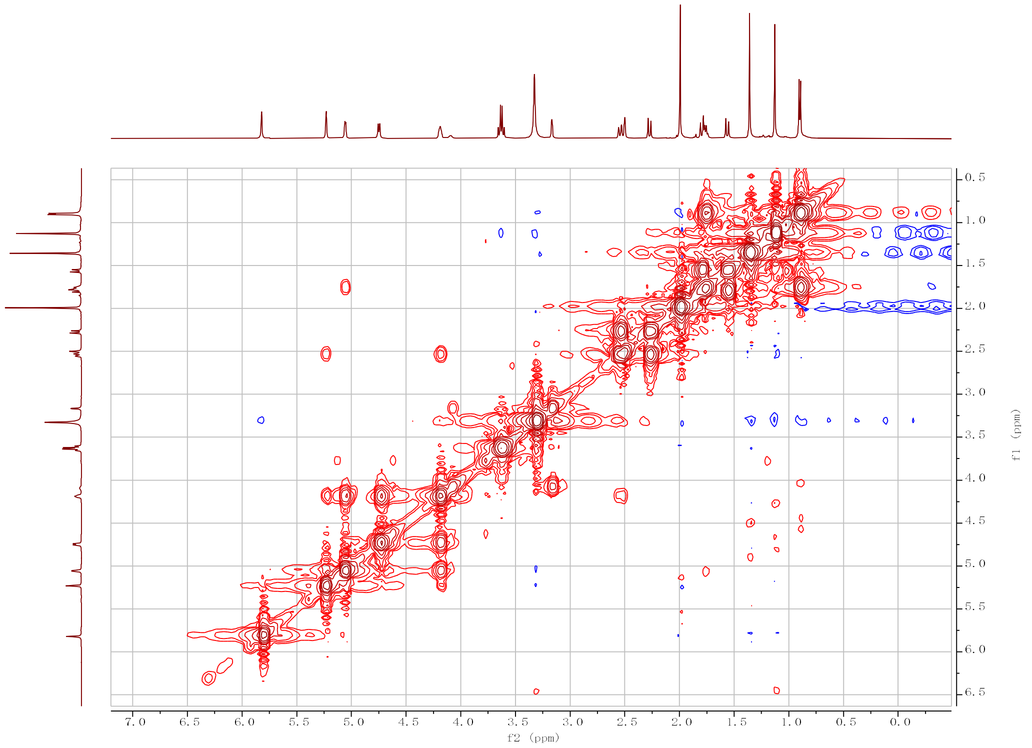

Figure S15. HSQC spectrum of compound **2**.

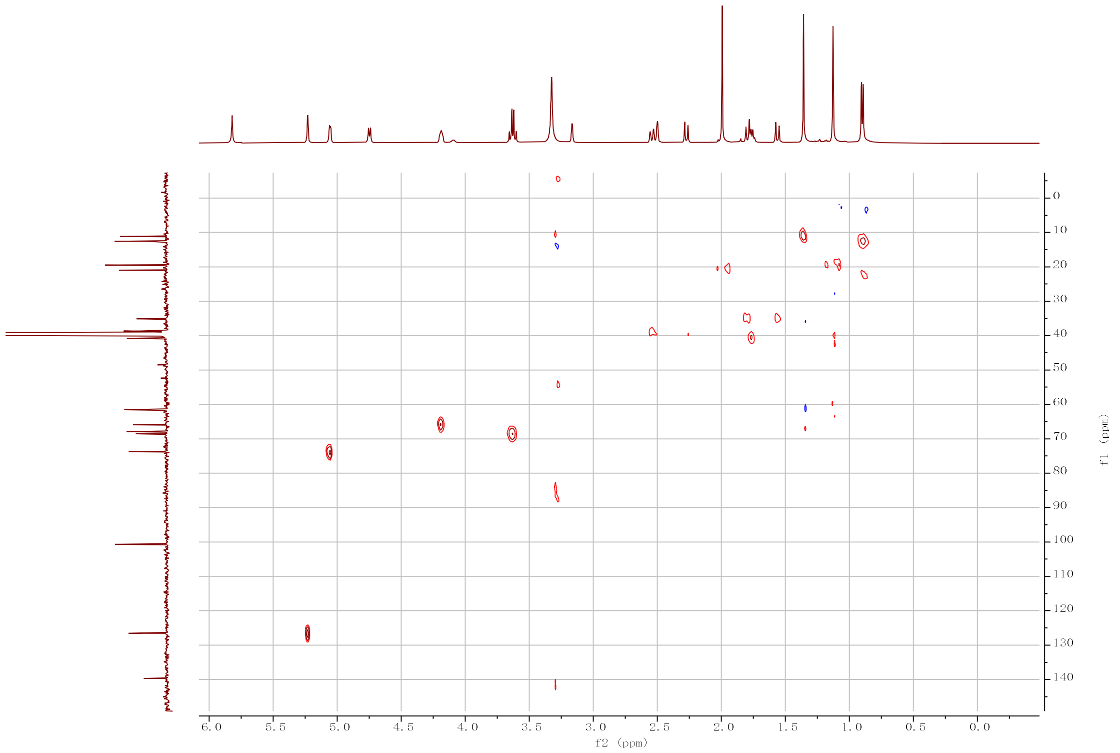

Figure S16. HMBC spectrum of compound **2**.

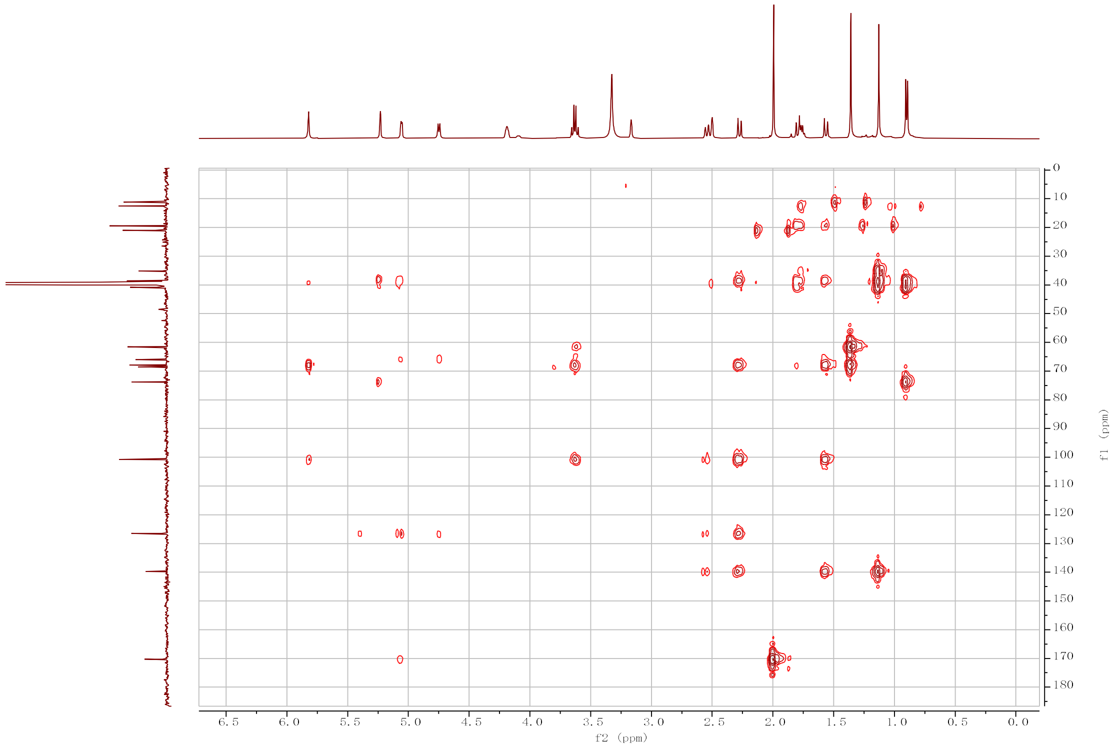

Figure S17. NOESY spectrum of compound **2**.

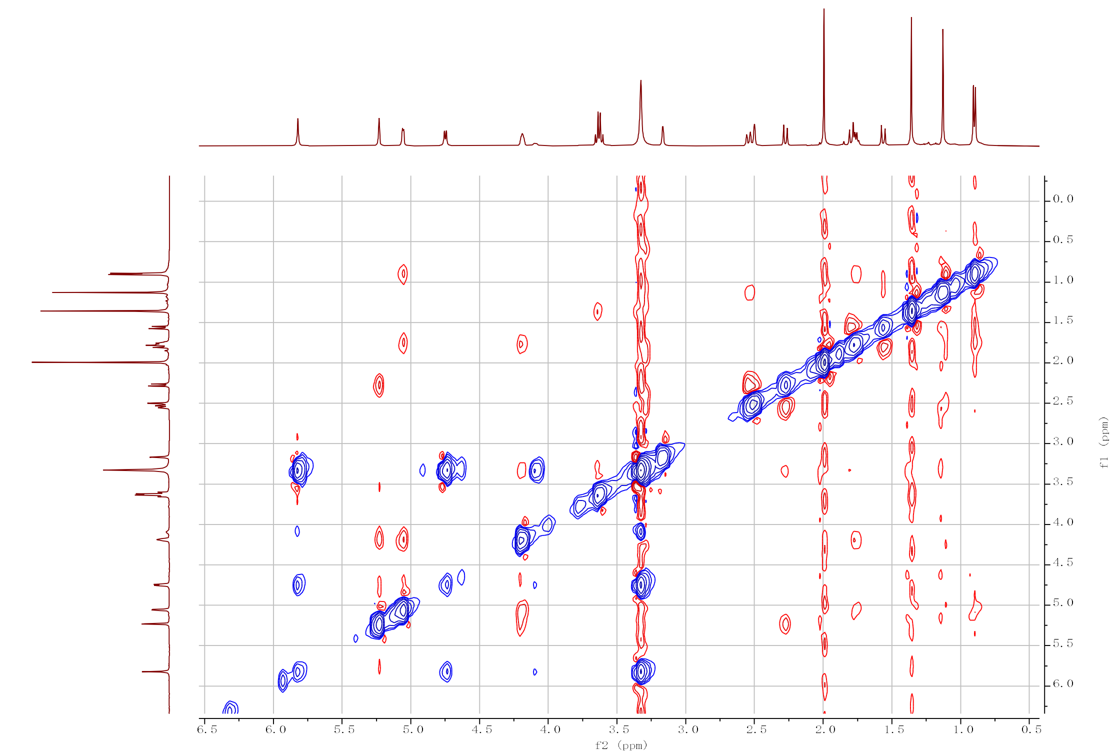

Figure S18. HRESI mass spectrum of compound **2**.

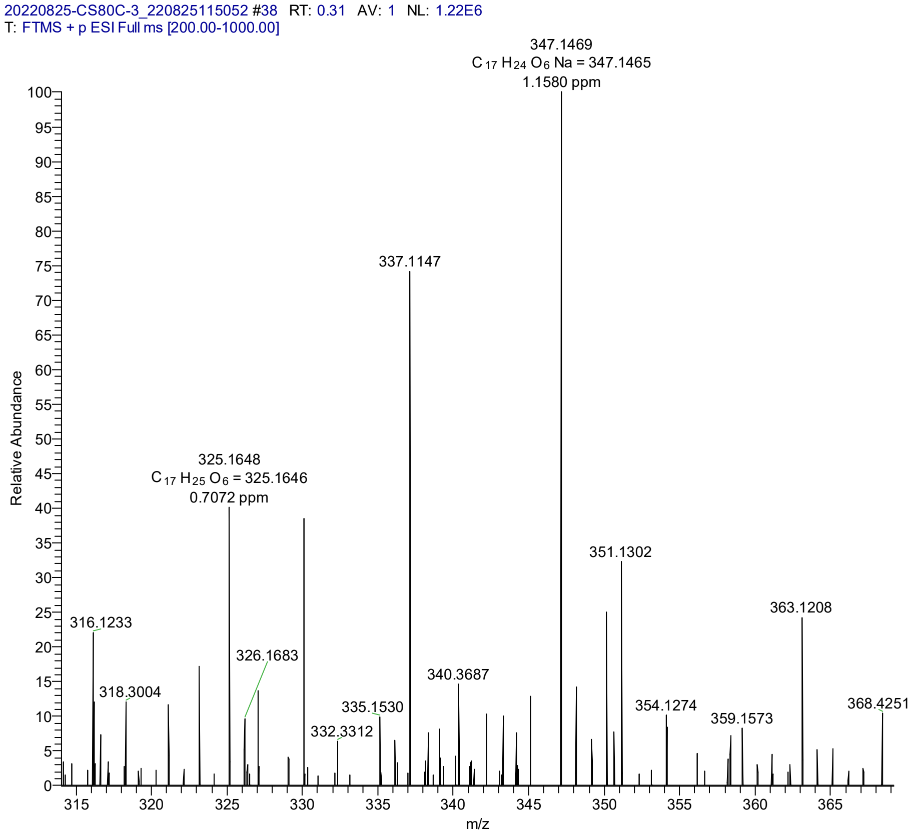

Figure S19. ECD spectrum of compound **2**.

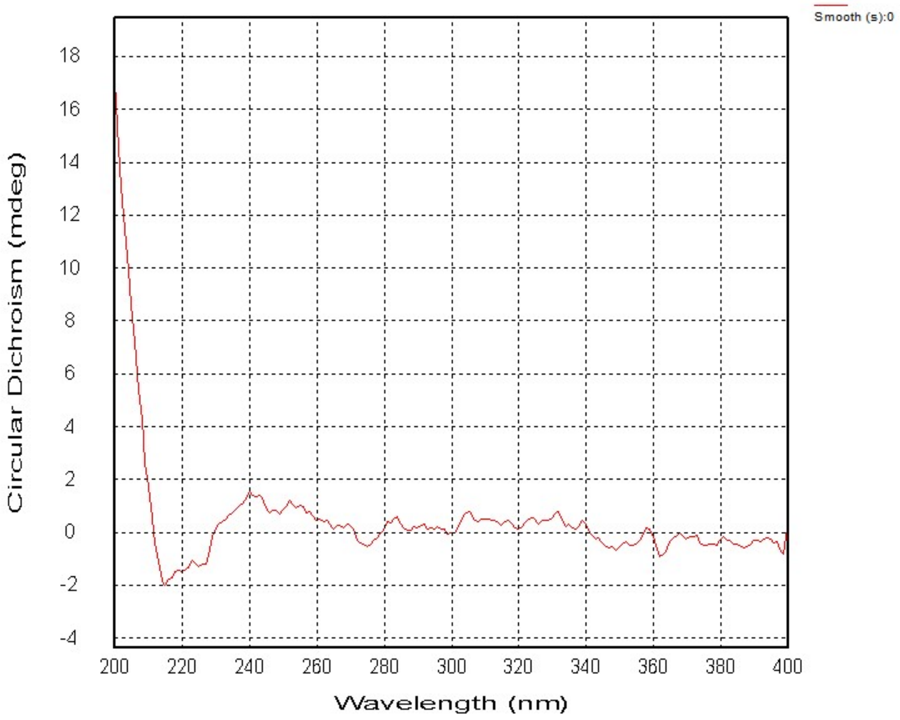

Figure S20. ^{1}H NMR (500 MHz, DMSO-d_6) spectrum of compound **3**.

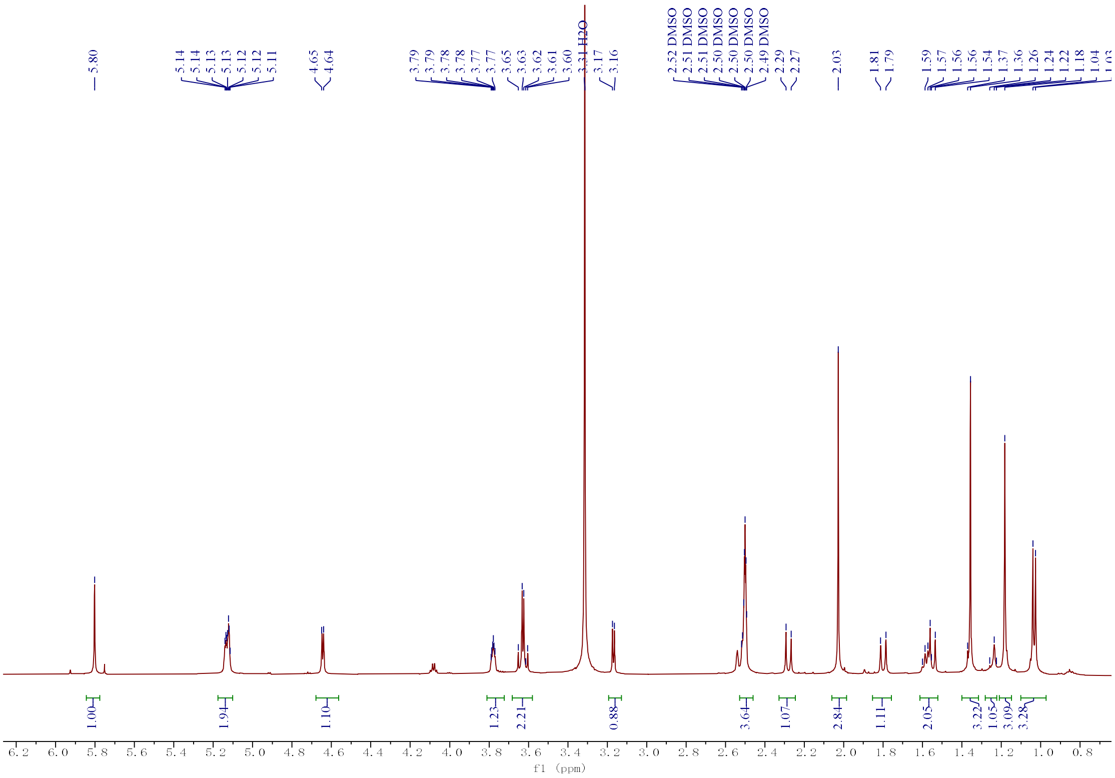

Figure S21.^{13}C NMR (125 MHz, DMSO-d_6) and DEPT spectra of compound **3**.

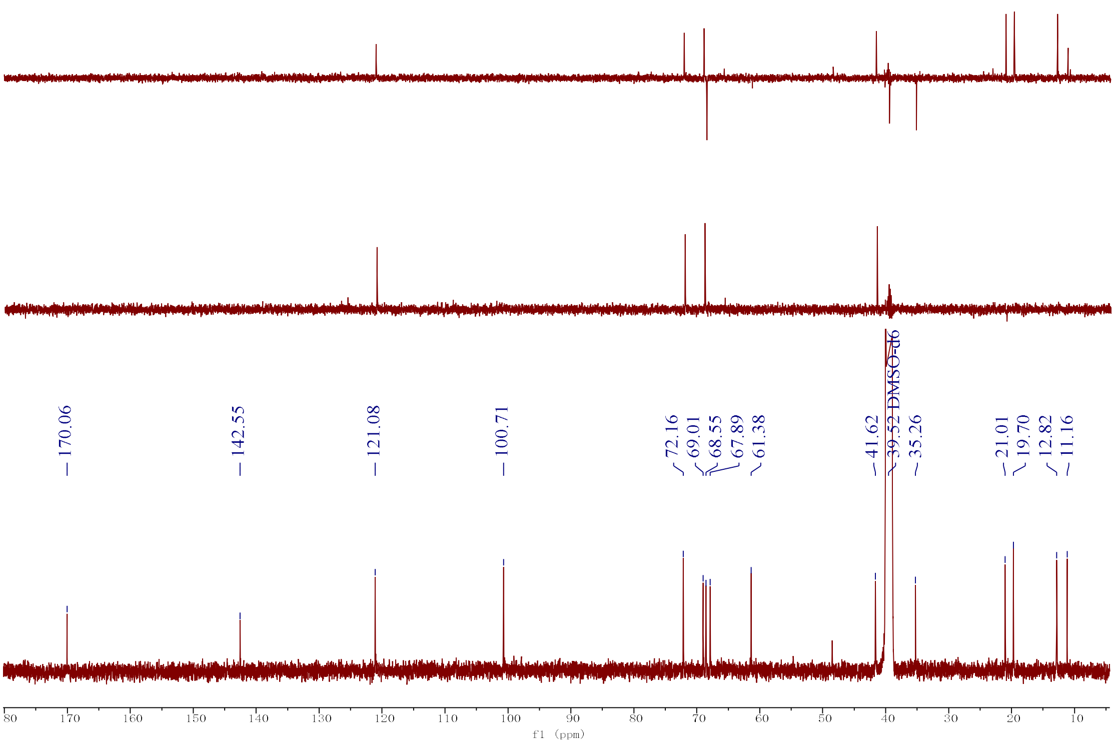

Figure S22. COSY spectrum of compound **3**.

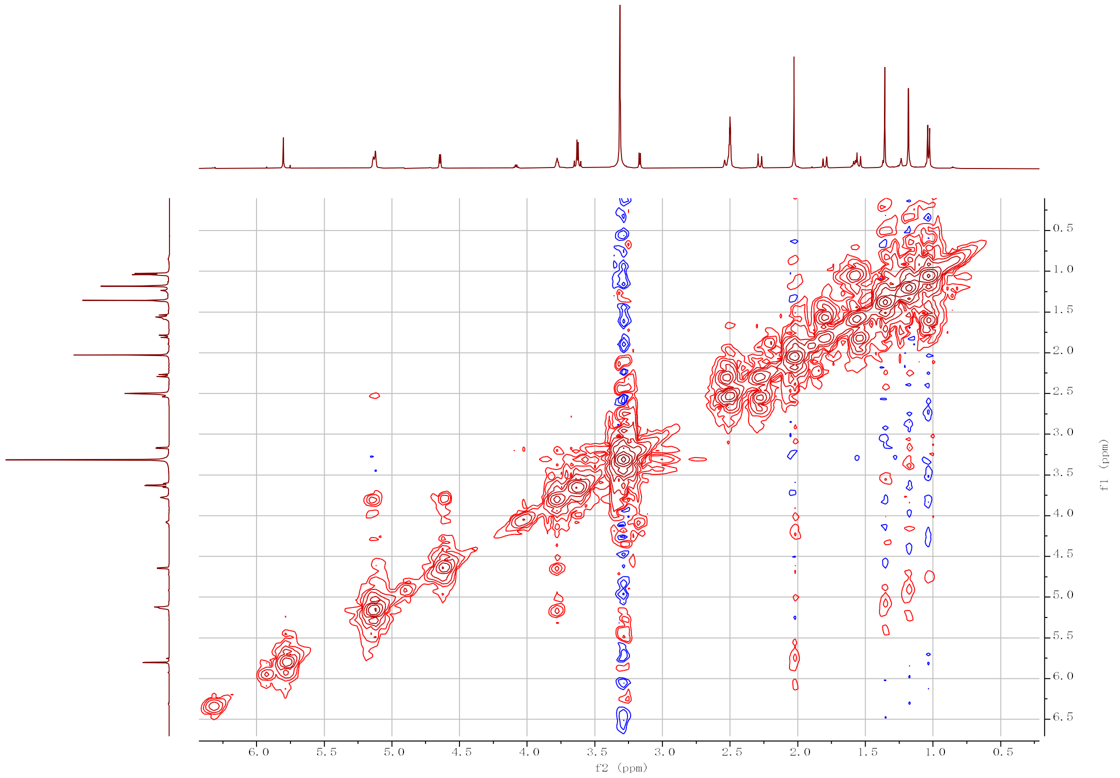

Figure S23. HSQC spectrum of compound **3**.

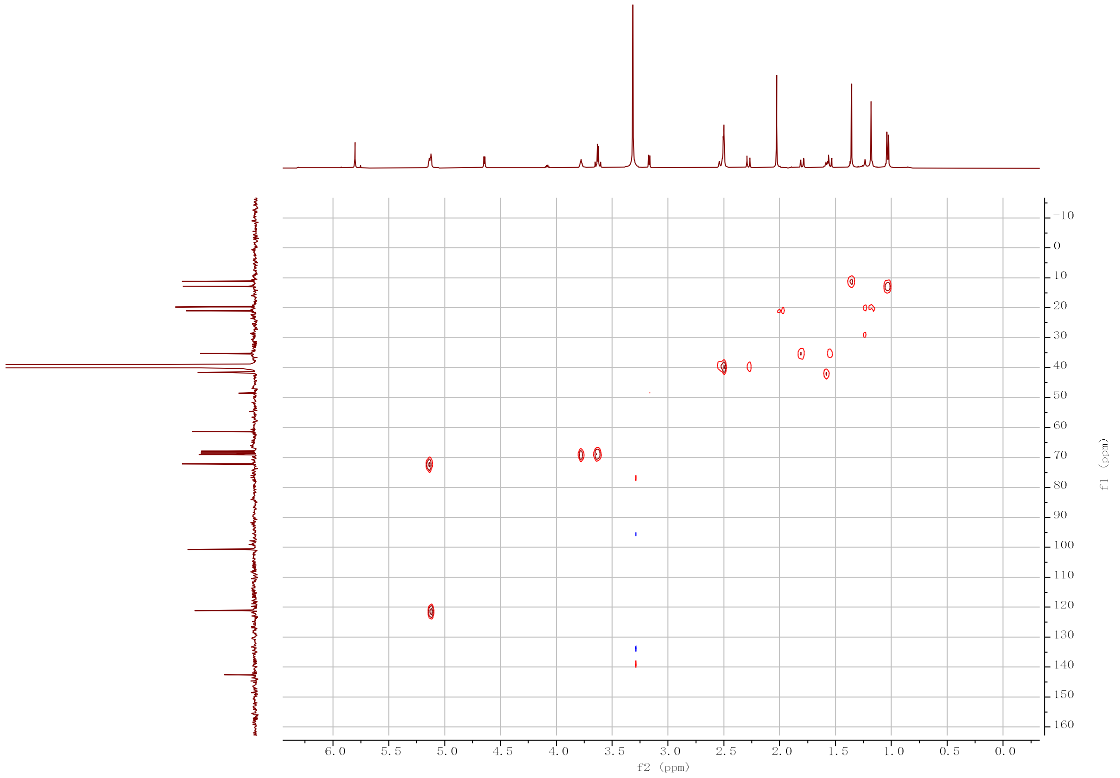

Figure S24. HMBC spectrum of compound **3**.

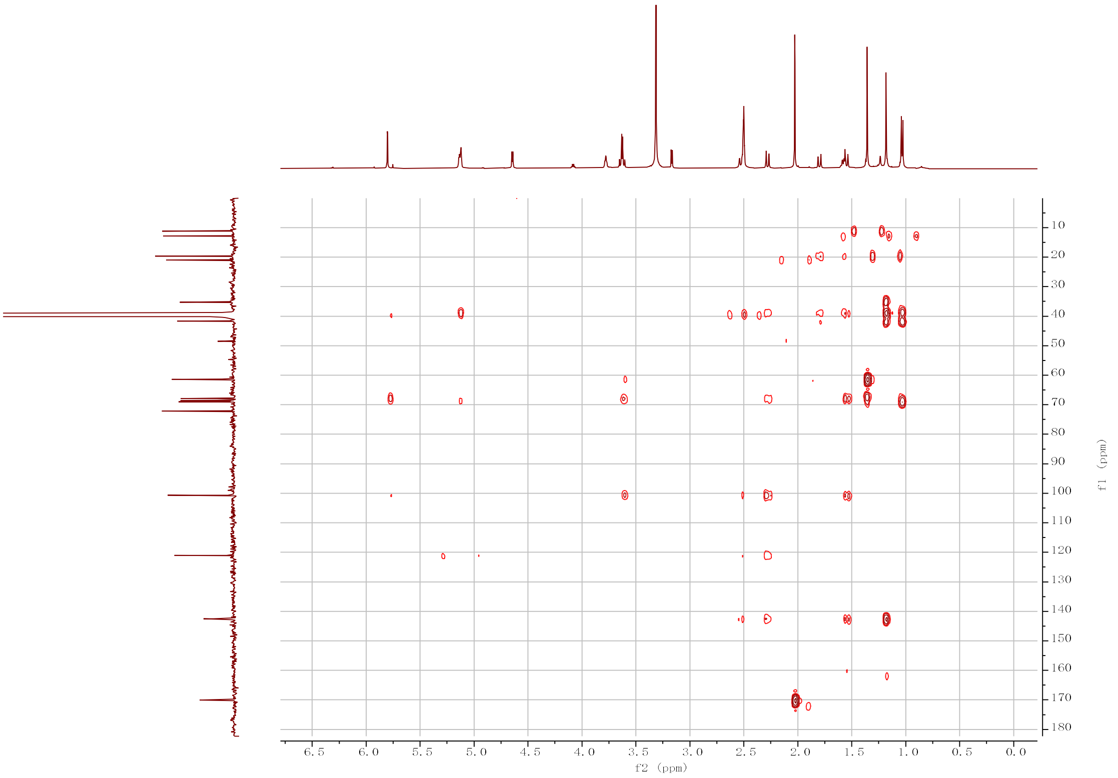

Figure S25. NOESY spectrum of compound **3**.

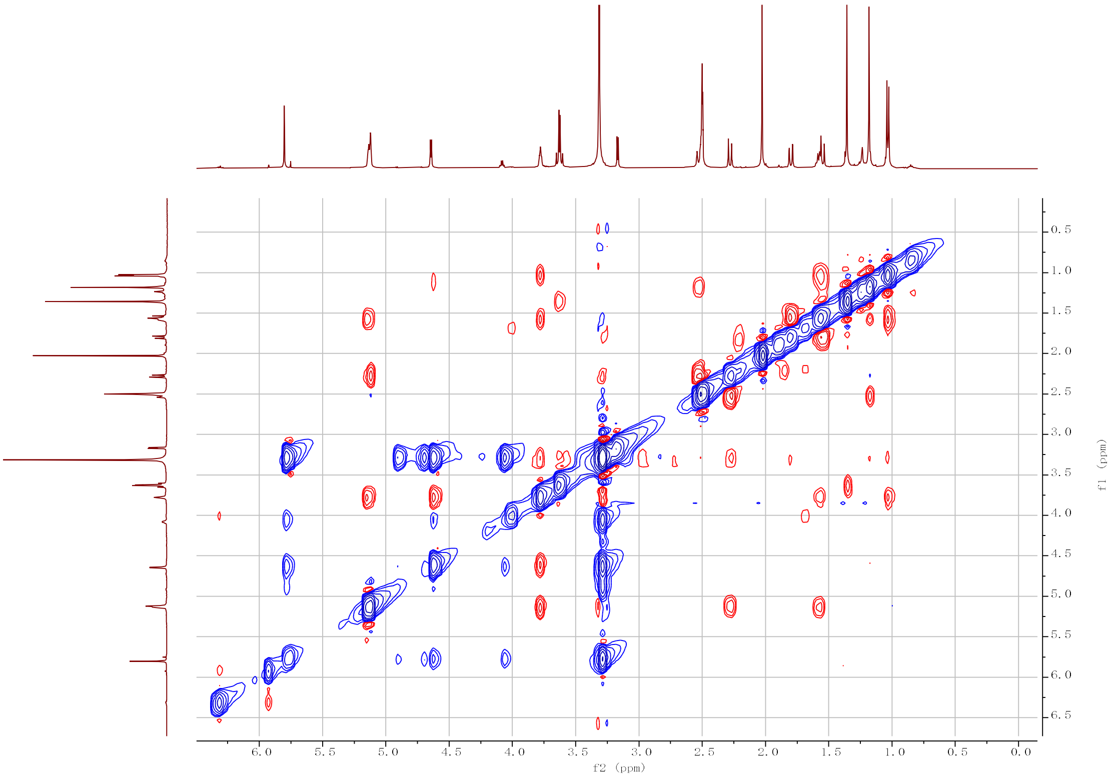

Figure S26. HRESI mass spectrum of compound **3**.

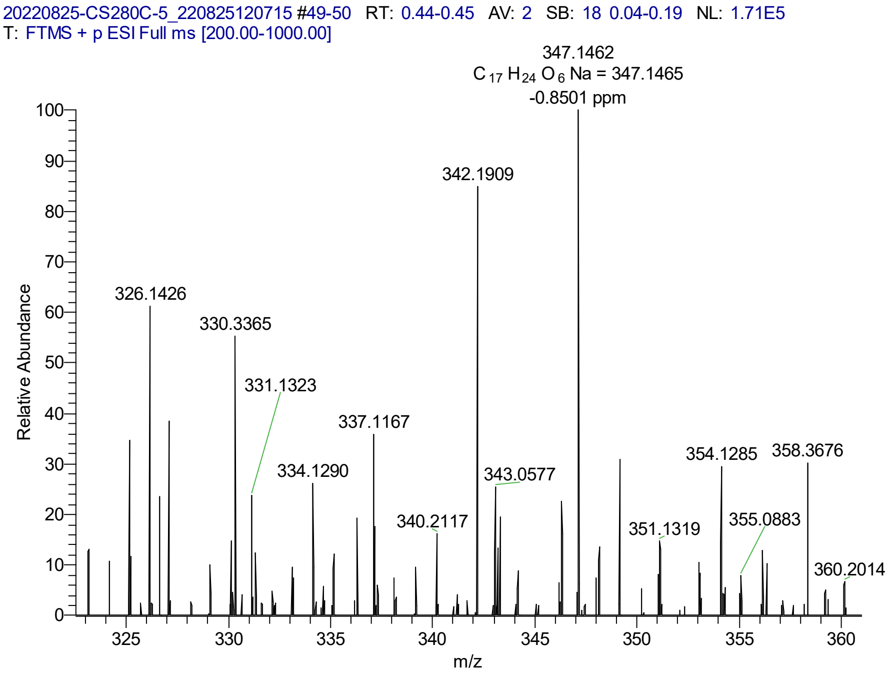

Figure S27. ECD spectrum of compound **3**

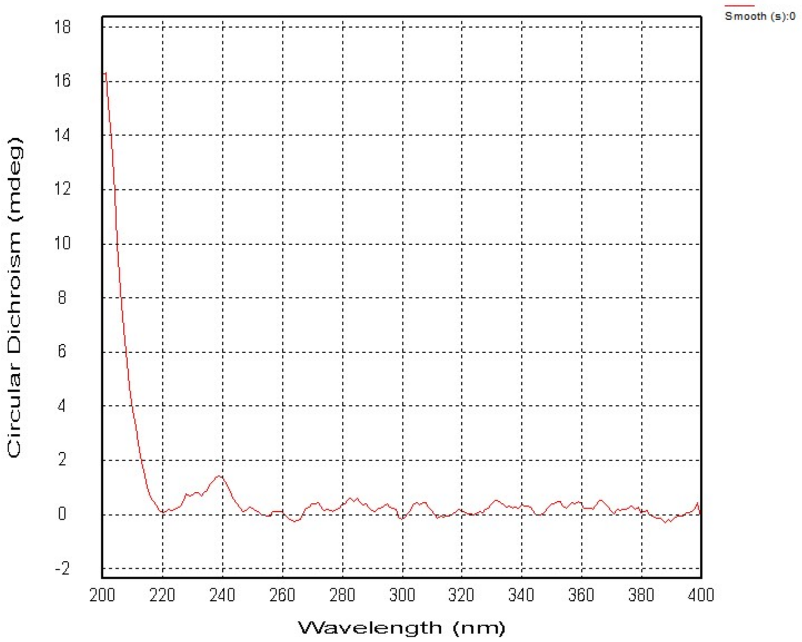

Figure S28. ^{1}H NMR (500 MHz, DMSO-d_6) spectrum of compound **4**.

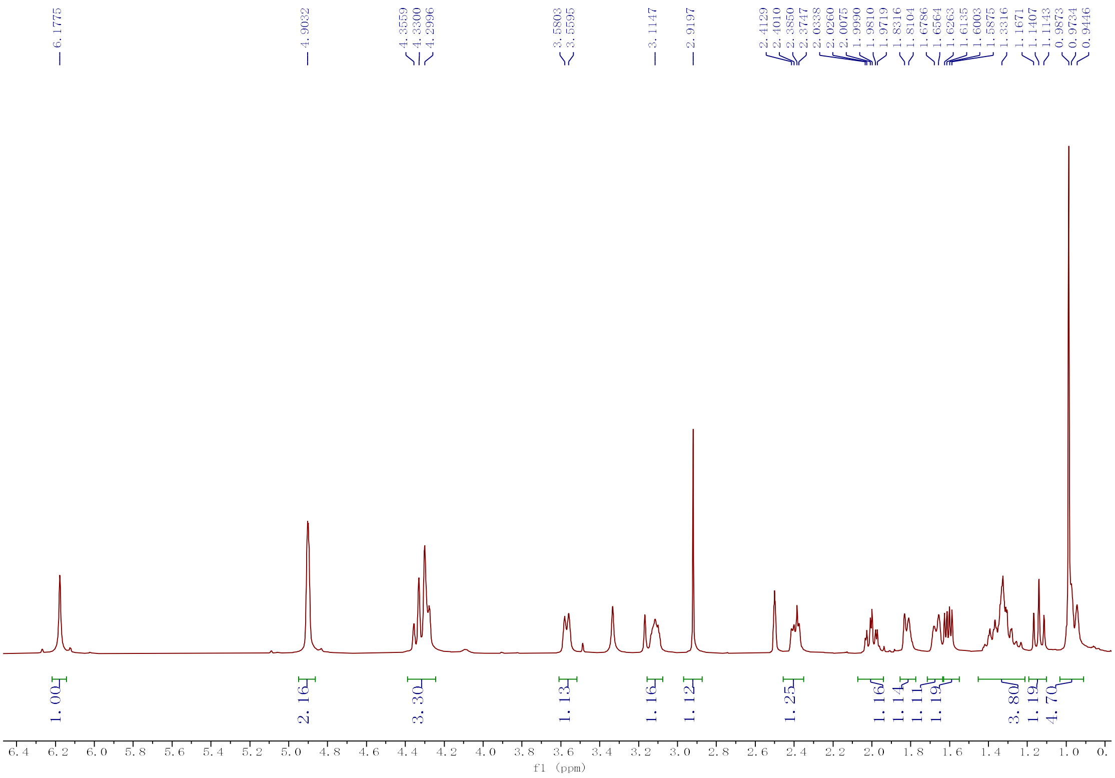

Figure S29. ^{13}C NMR (125 MHz, DMSO-d_6) and DEPT spectra of compound **4**.

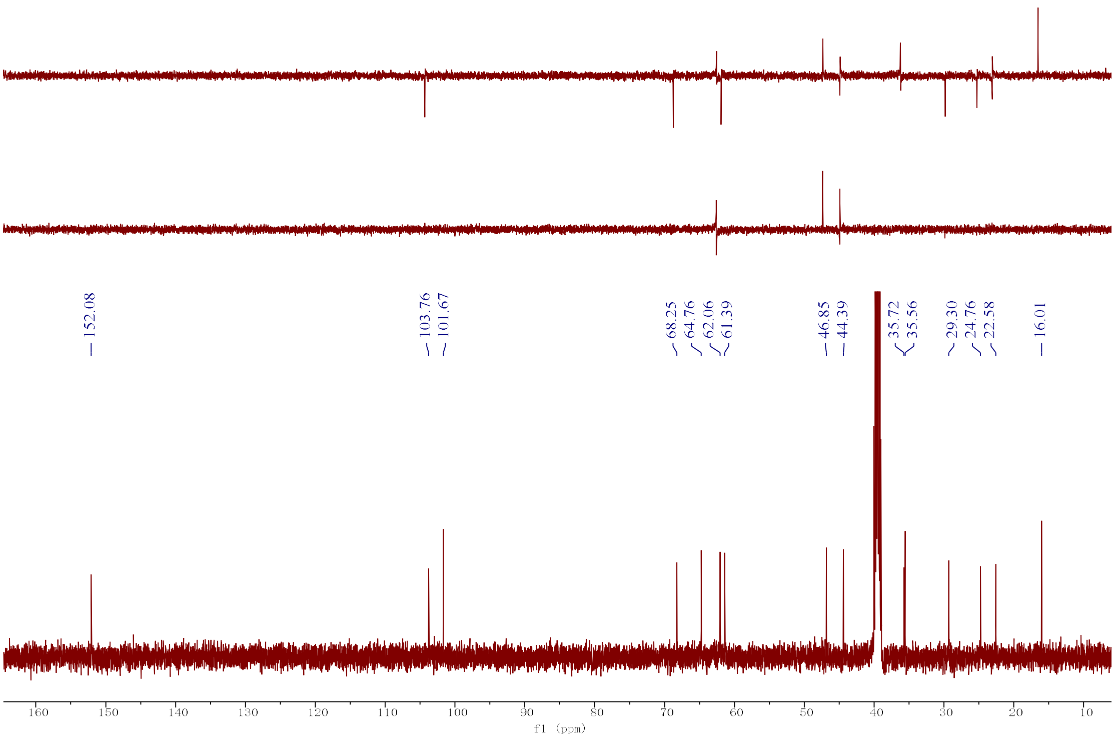

Figure S30. COSY spectrum of compound **4**.

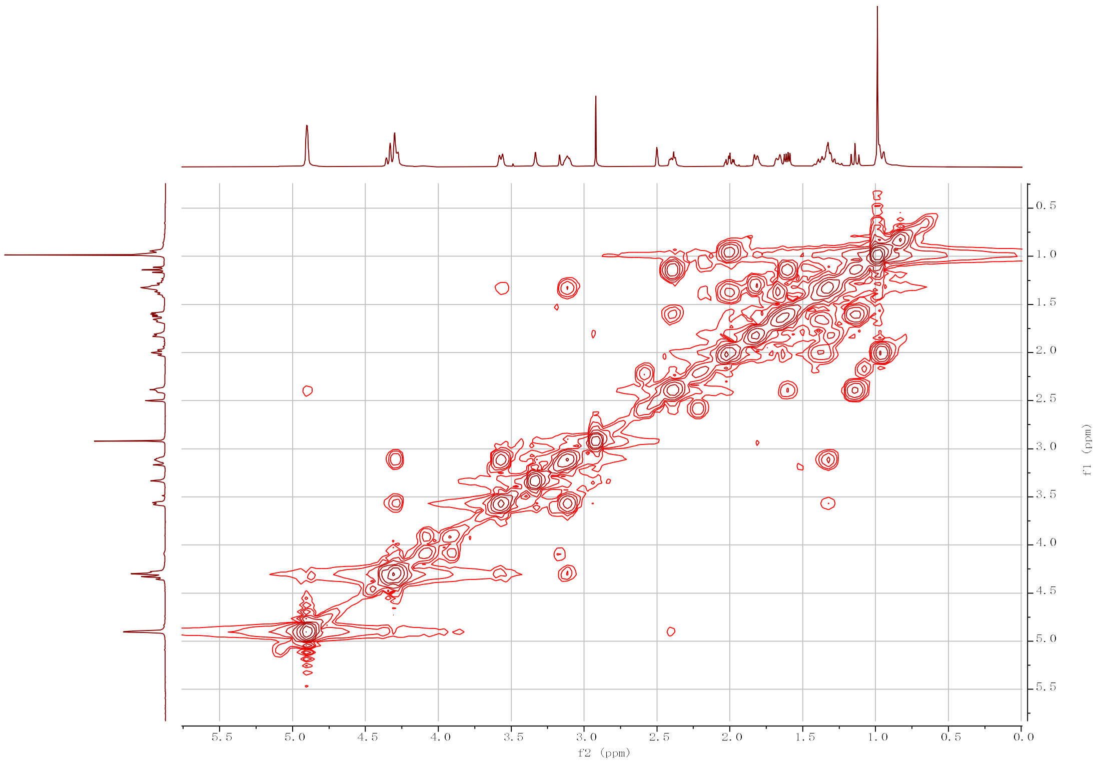

Figure S31. HSQC spectrum of compound **4**.

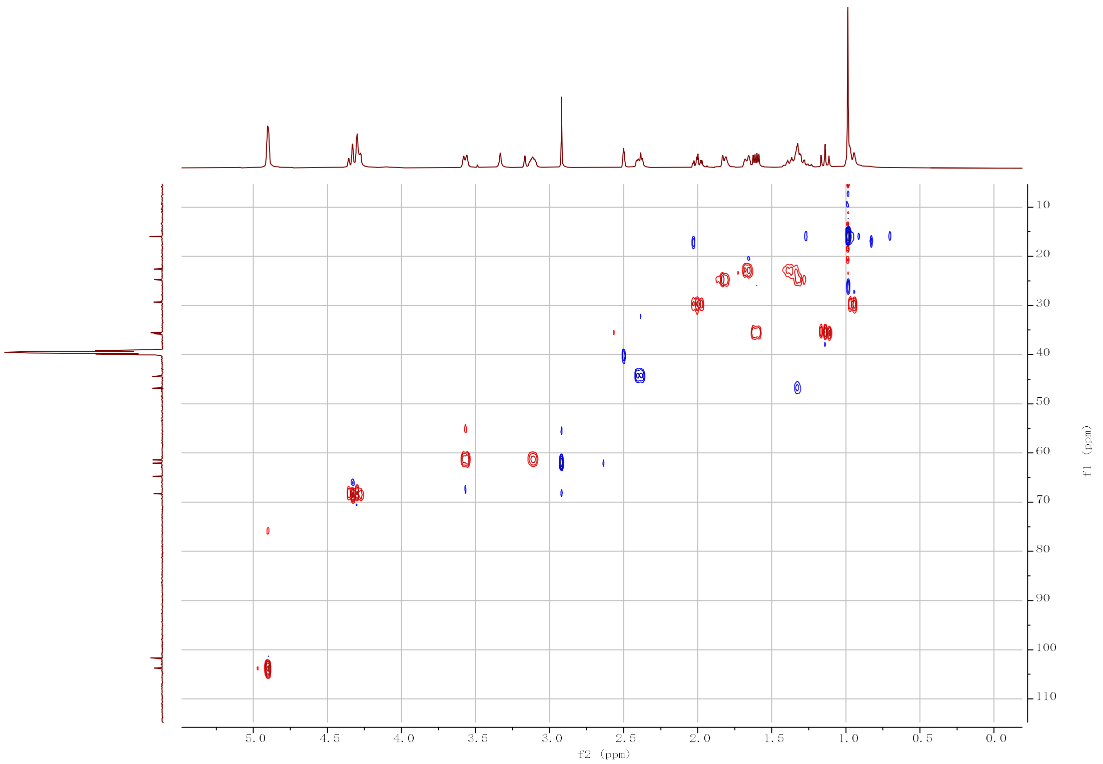

Figure S32. HMBC spectrum of compound **4**.

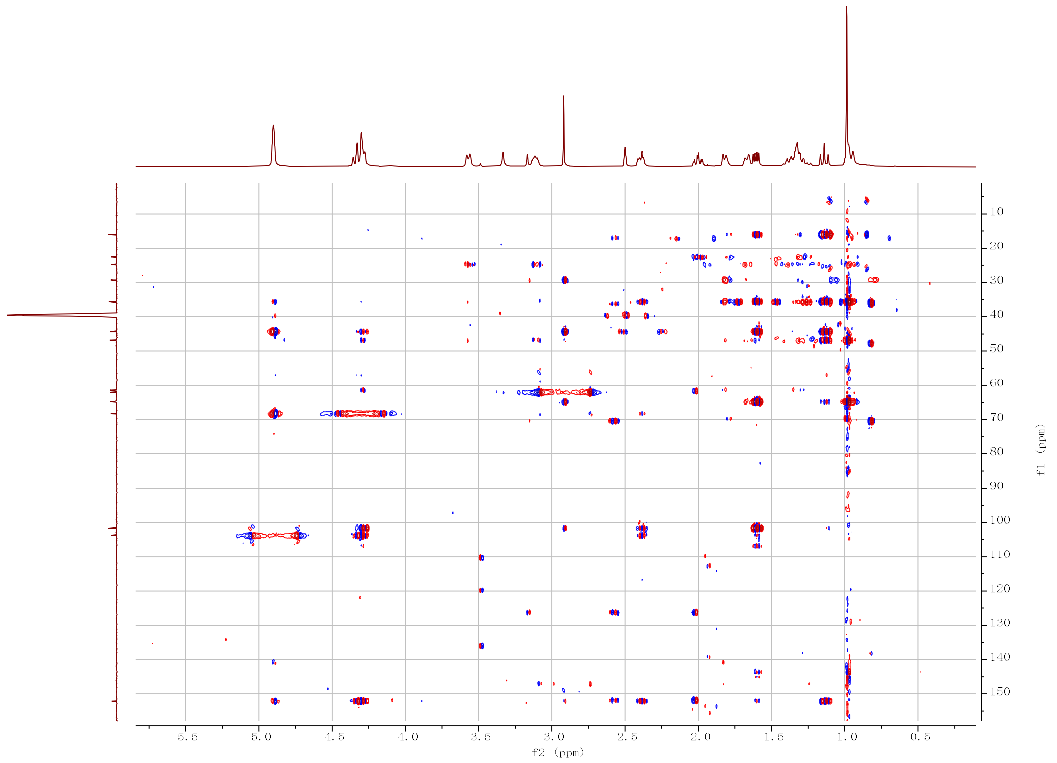

Figure S33. NOESY spectrum of compound **4**.

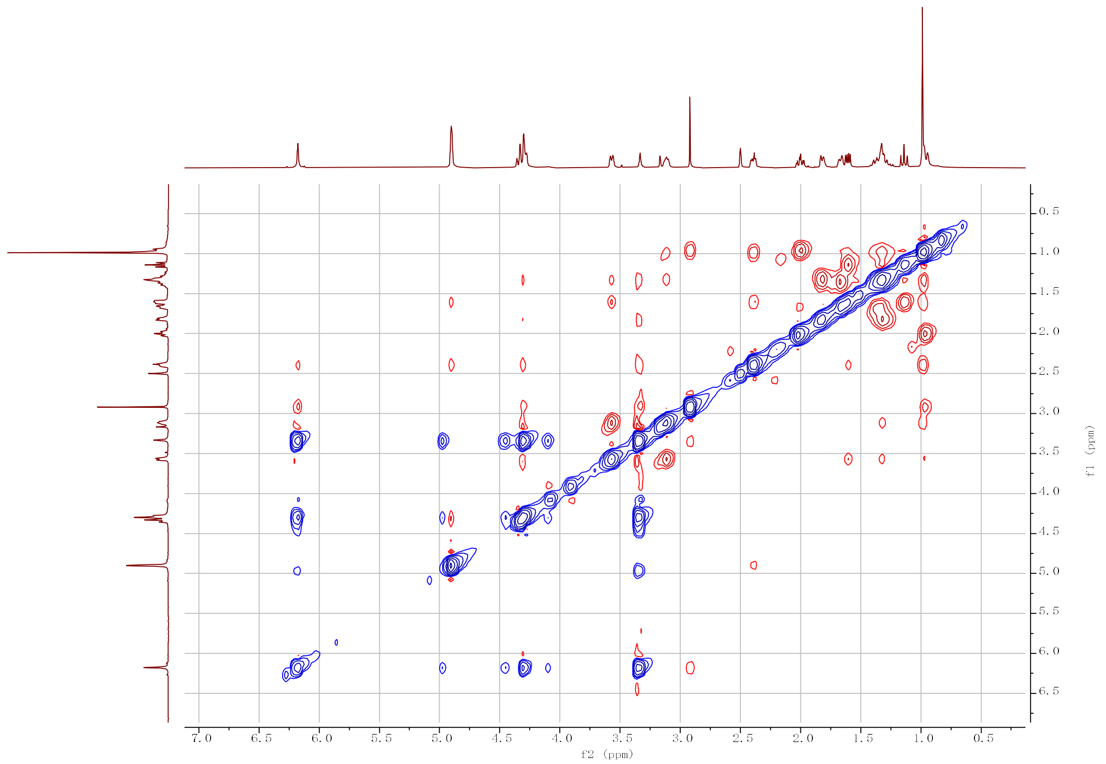

Figure S34. HRESI mass spectrum of compound **4**.

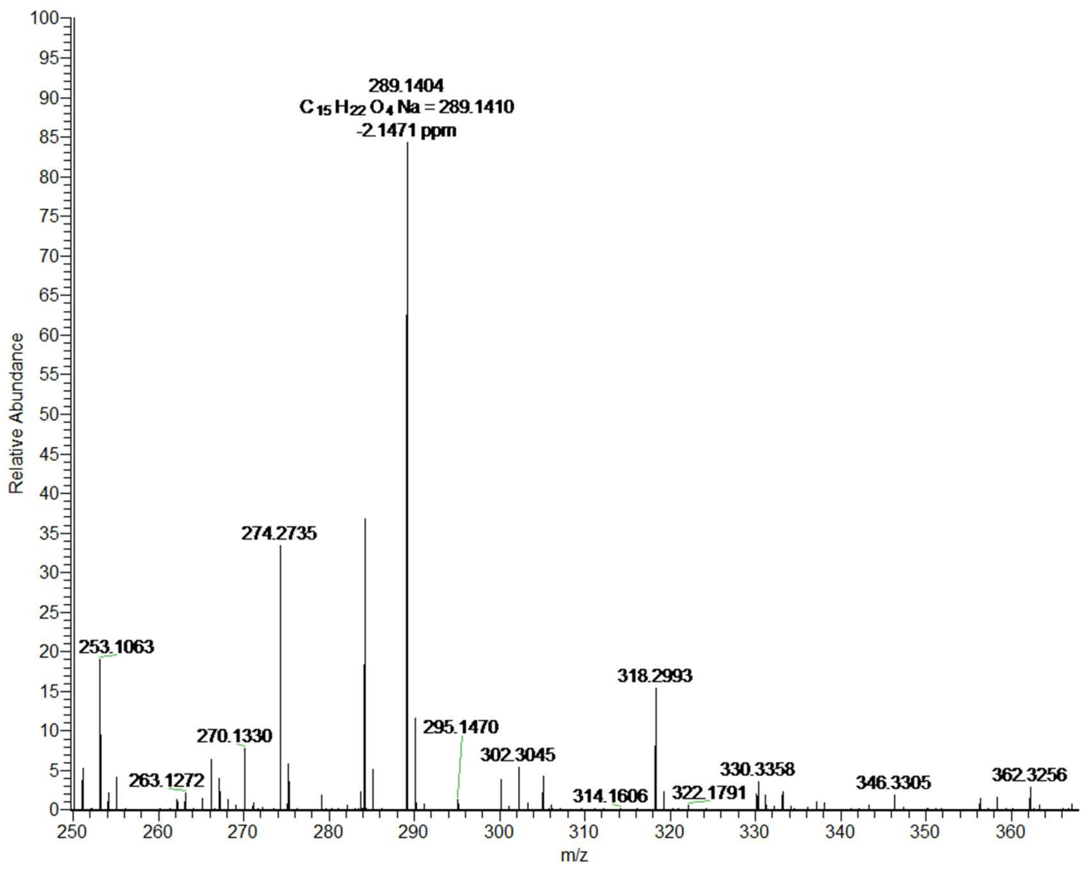

Figure S35. ^{1}H NMR (500 MHz, DMSO-d_6) spectrum of compound **5**.

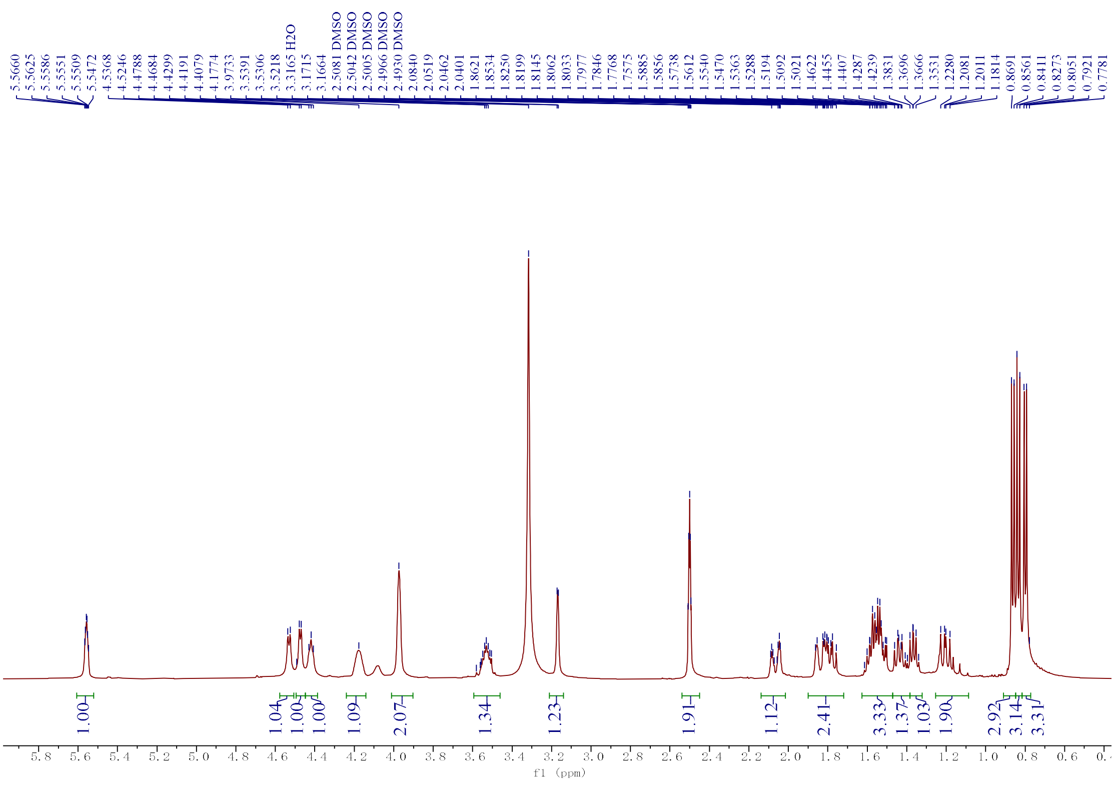

Figure S36.^{13}C NMR (125 MHz, DMSO-d_6) and DEPT spectra of compound **5**.

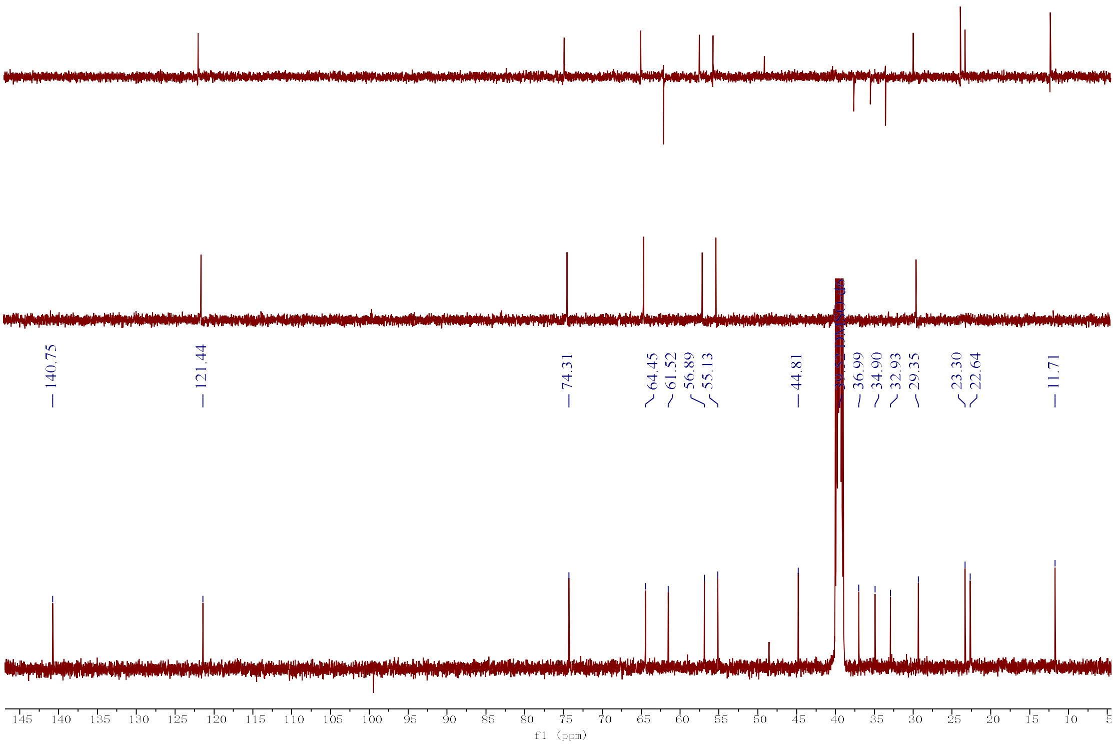

Figure S37. COSY spectrum of compound **5**.

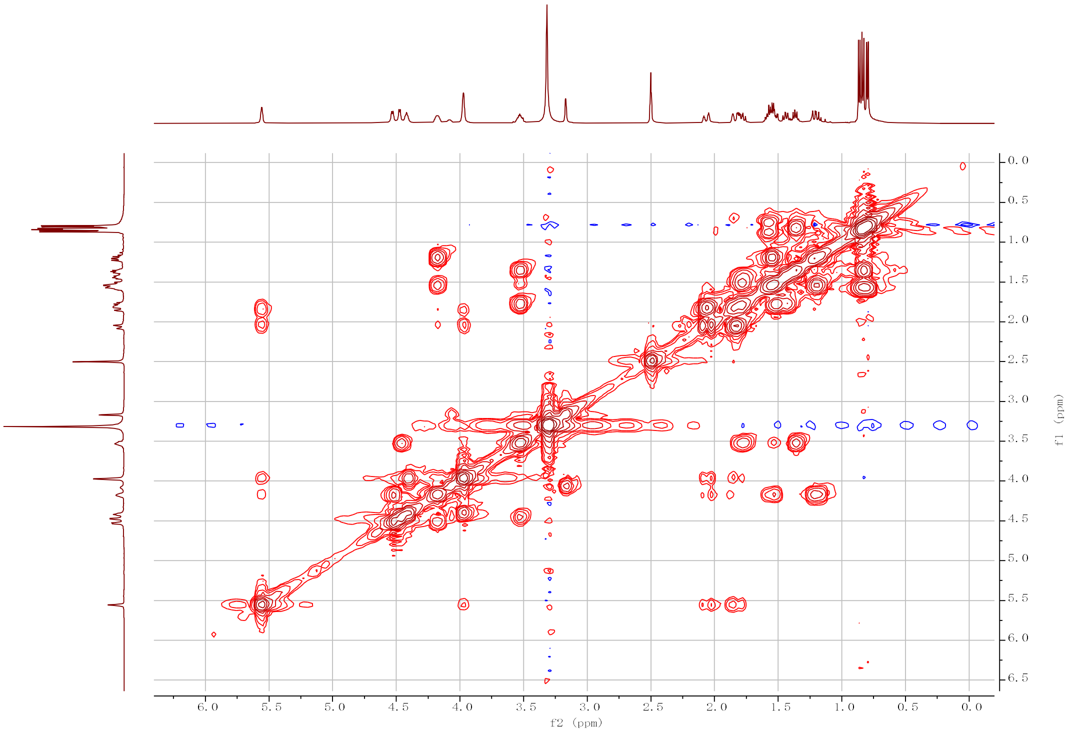

Figure S38. HSQC spectrum of compound **5**.

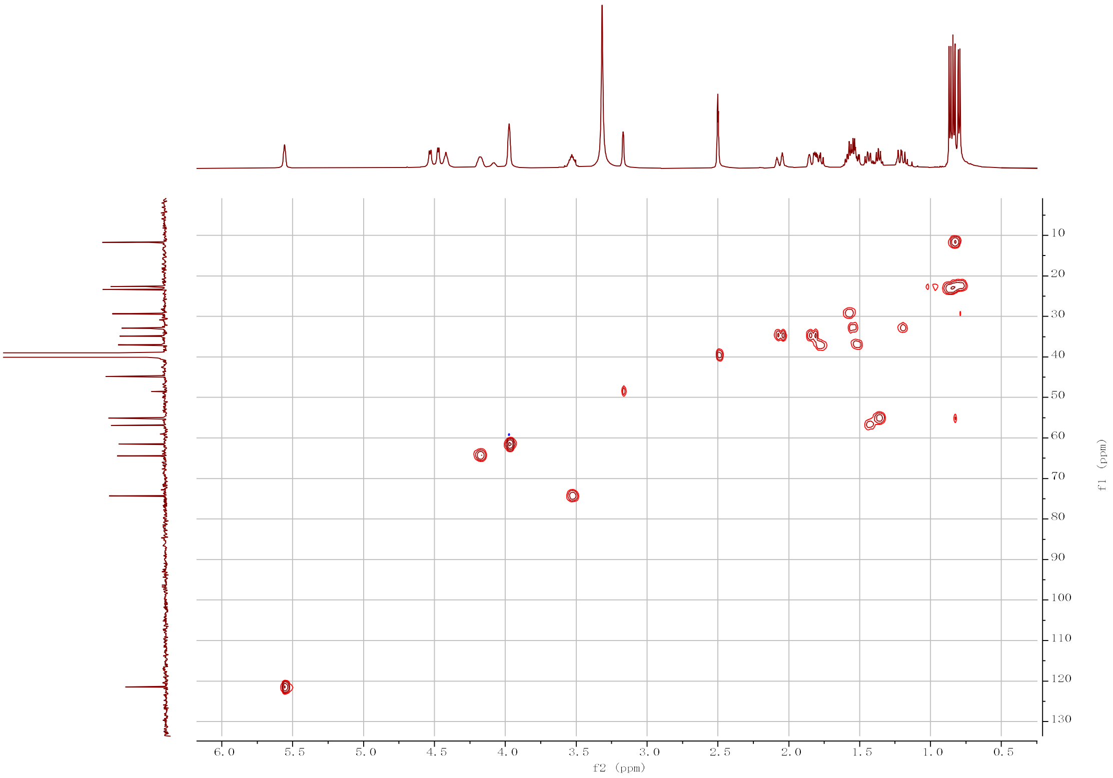

Figure S39. HMBC spectrum of compound **5**.

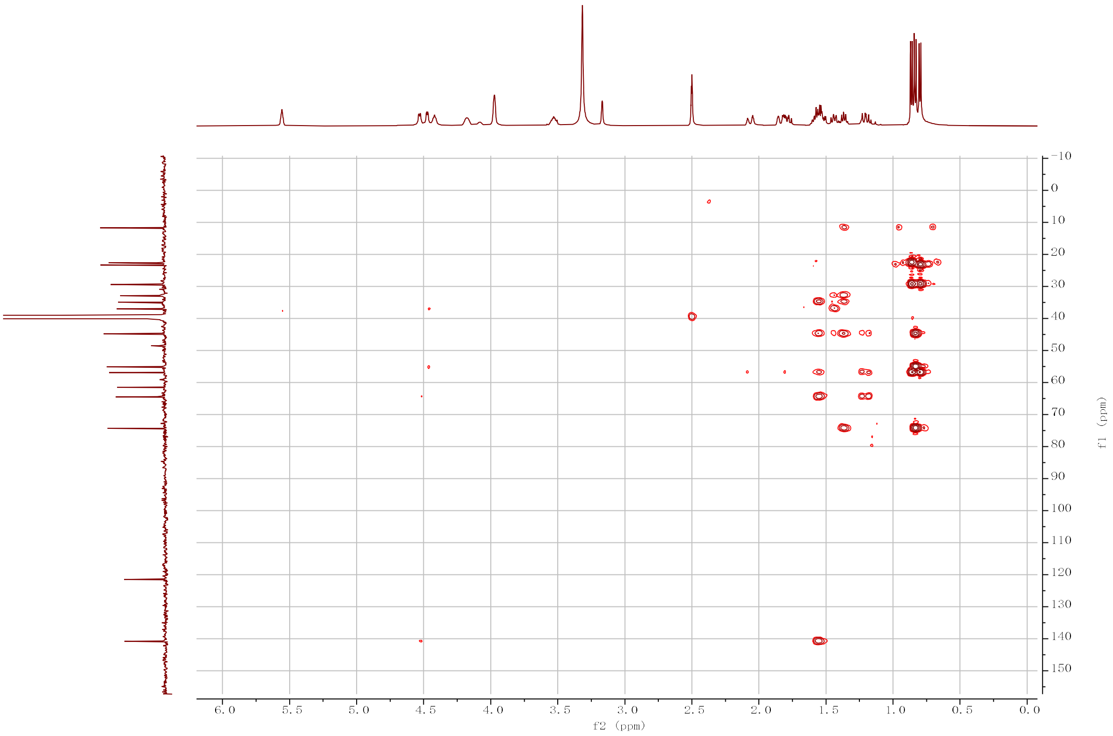

Figure S40. NOESY spectrum of compound **5**.

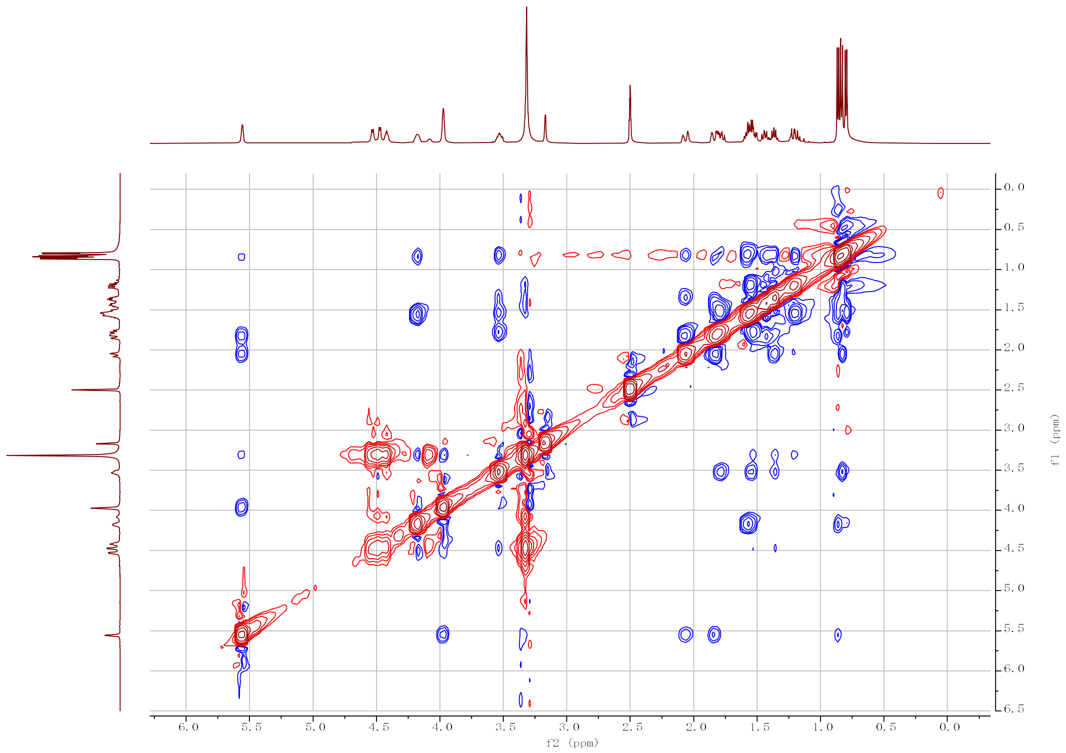

Figure S41. ^{1}H NMR (600 MHz, $CDCl_3$) of compound **5** in $CDCl_3$.

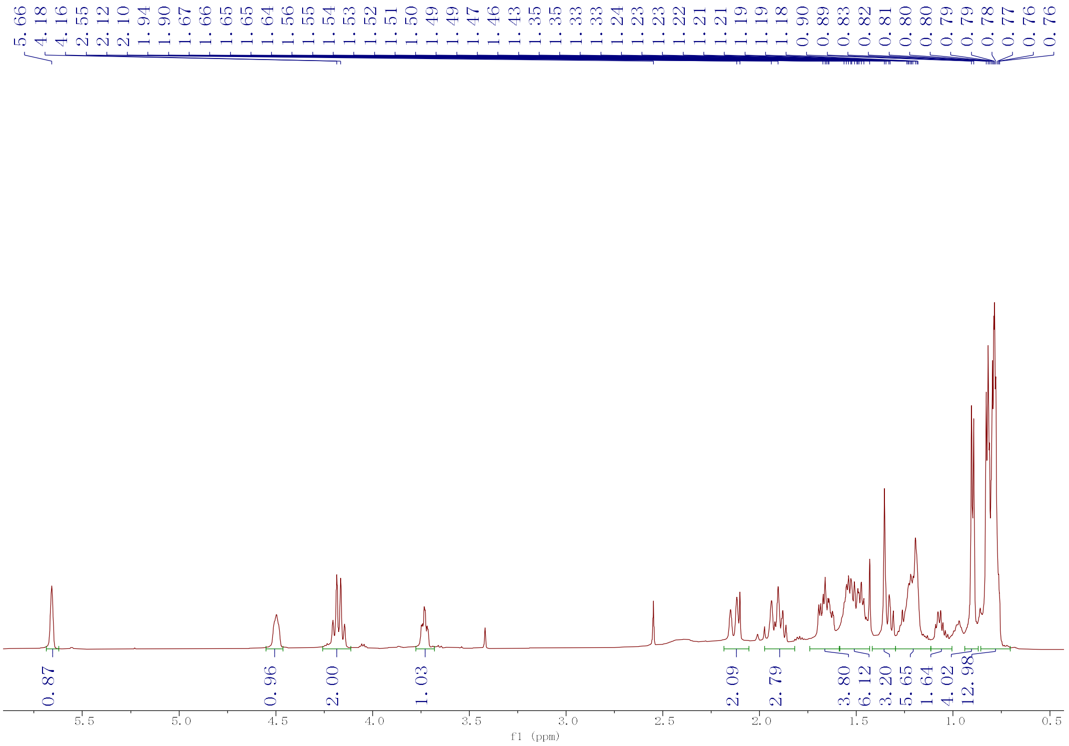

Figure S42. HSQC spectrum of compound **5** in $CDCl_3$.

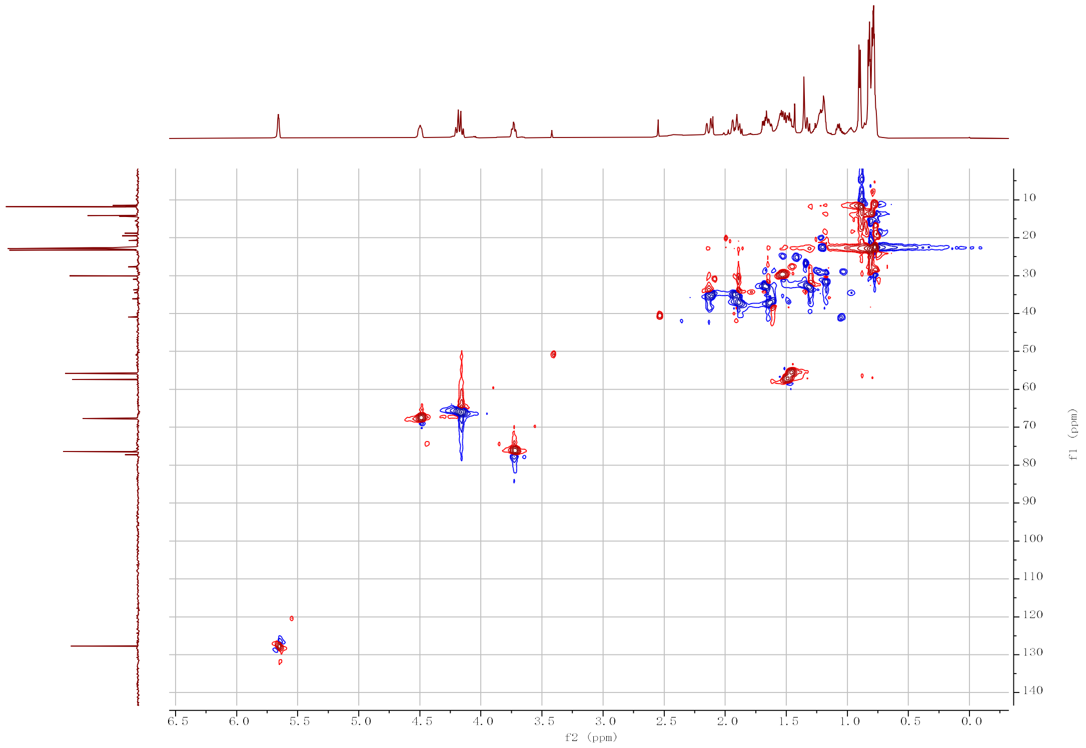

Figure S43. NOESY spectrum of compound **5** in $CDCl_3$.

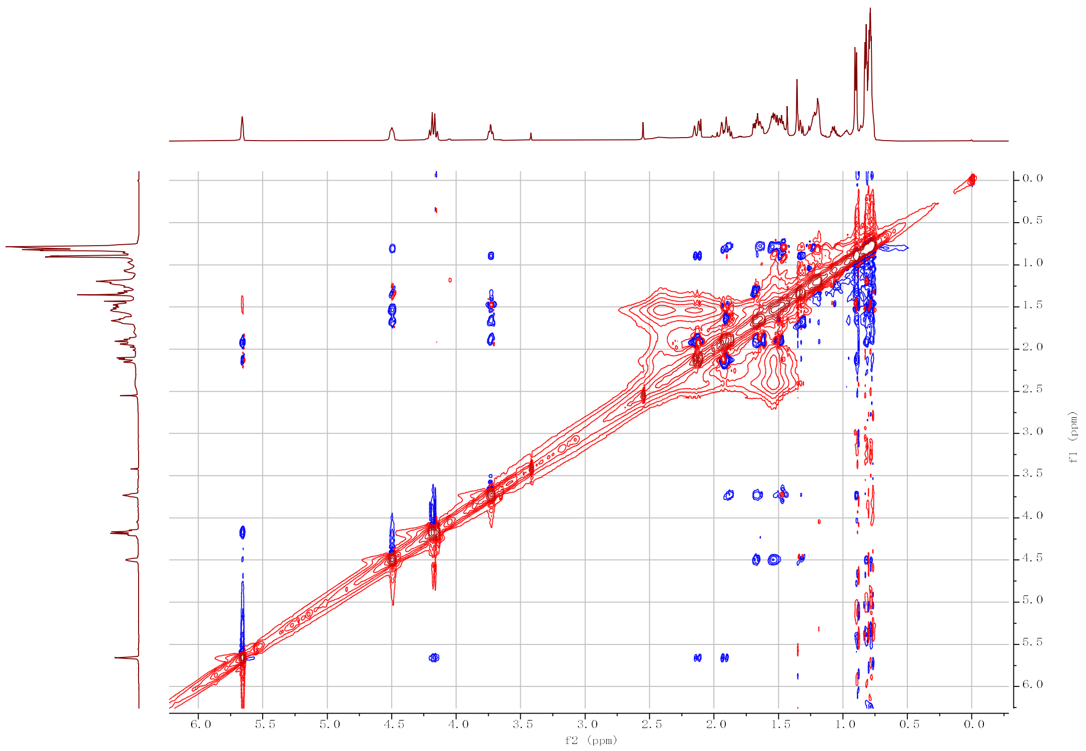

Figure S44. HRESI mass spectrum of compound **5**.

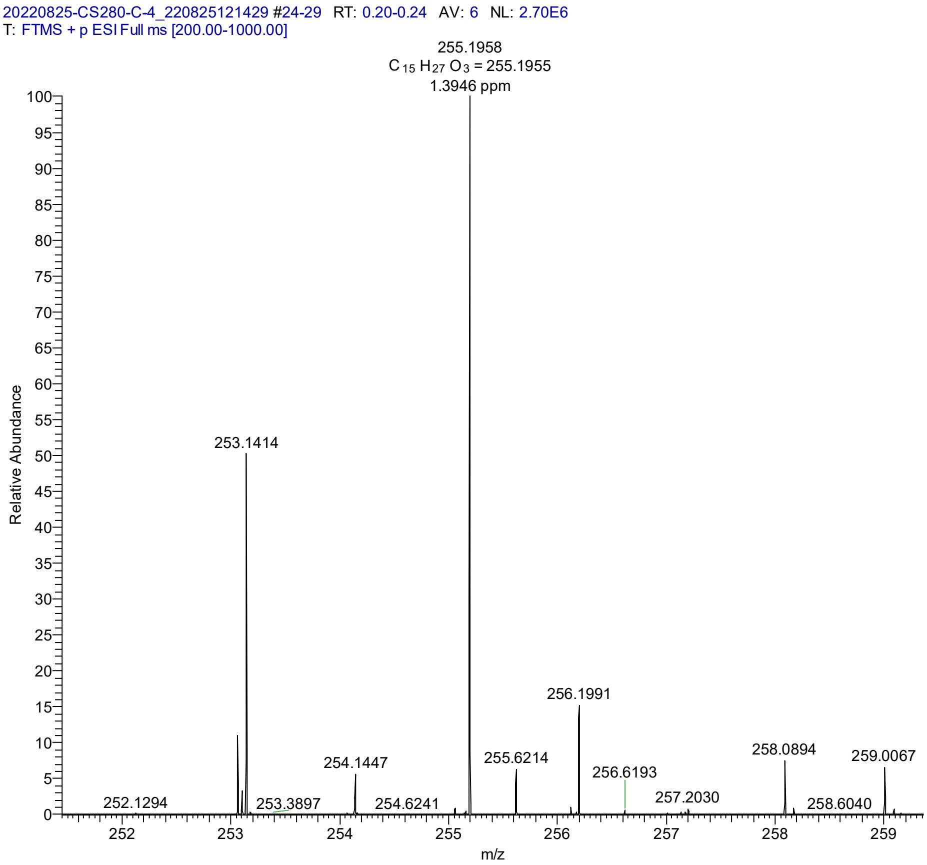

Figure S45. ^{1}H NMR (500 MHz, DMSO-d_6) spectrum of compound **6**.

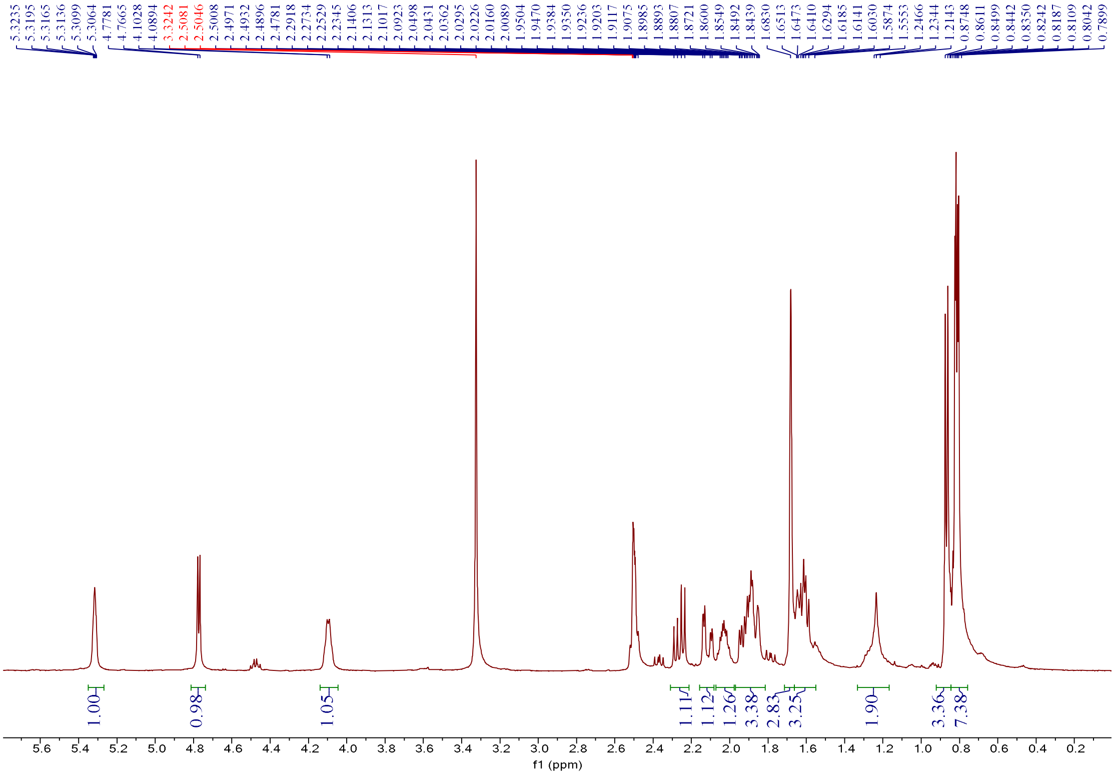

Figure S46. ^{13}C NMR (125 MHz, DMSO-d_6) and DEPT spectra of compound **6**.

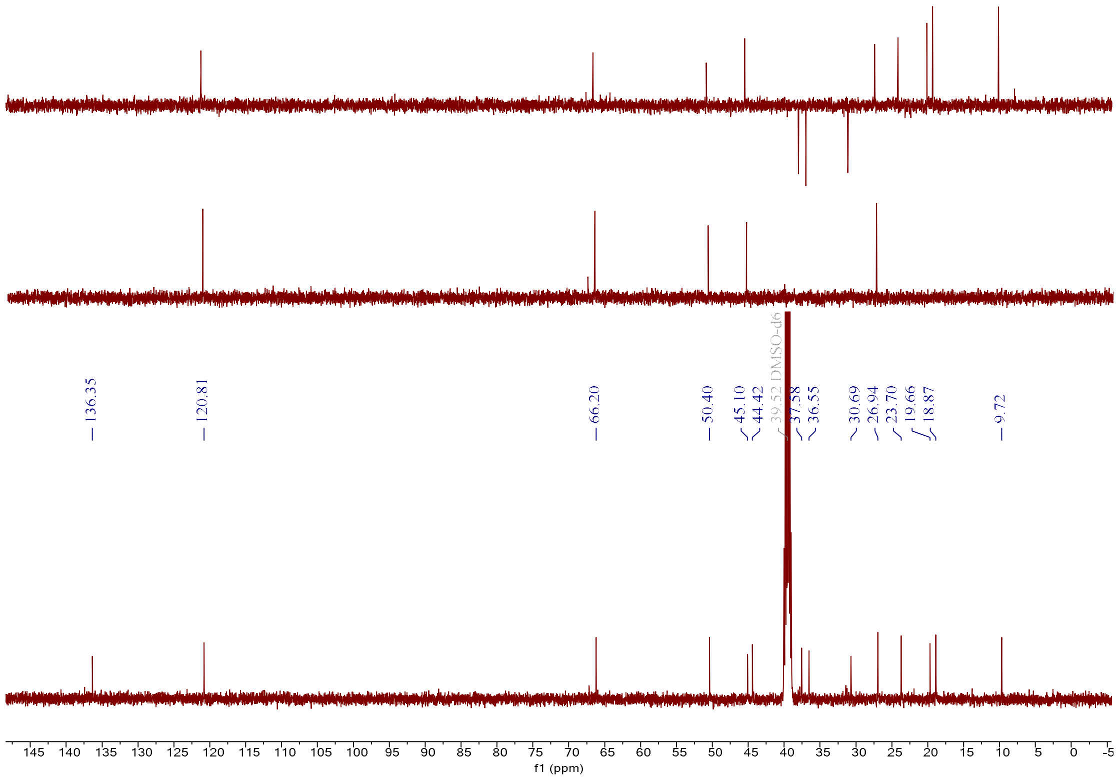

Figure S47. COSY spectrum of compound **6**.

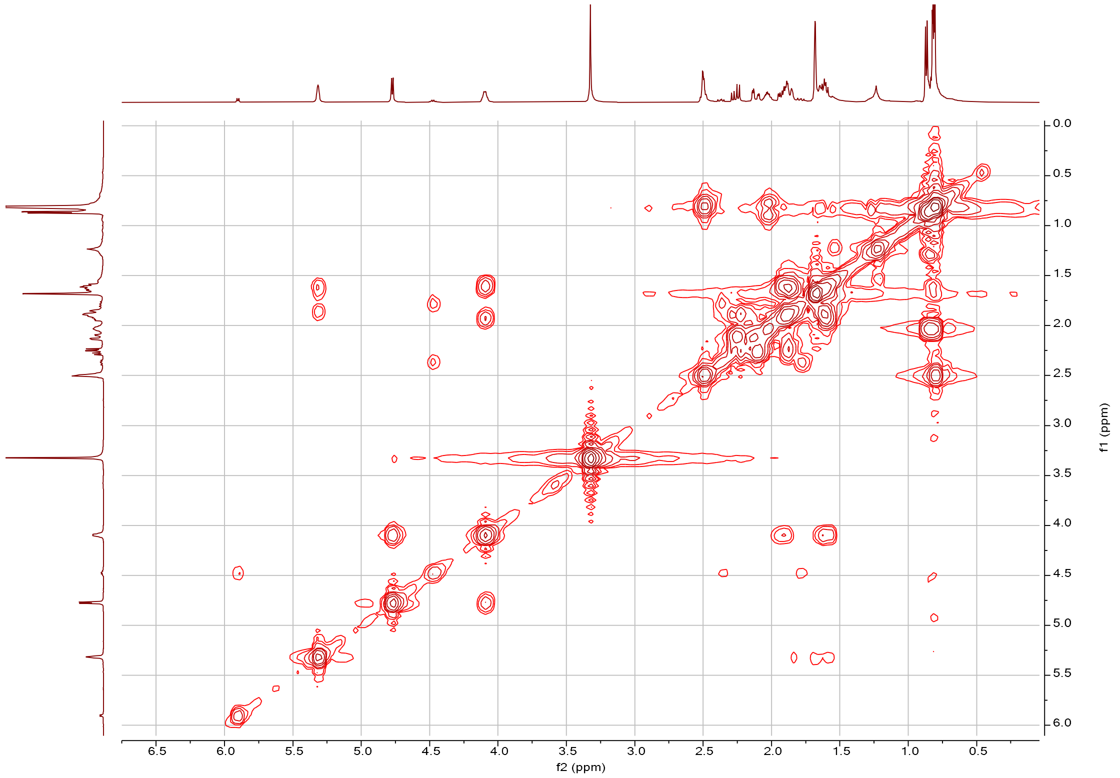

Figure S48. HSQC spectrum of compound **6**.

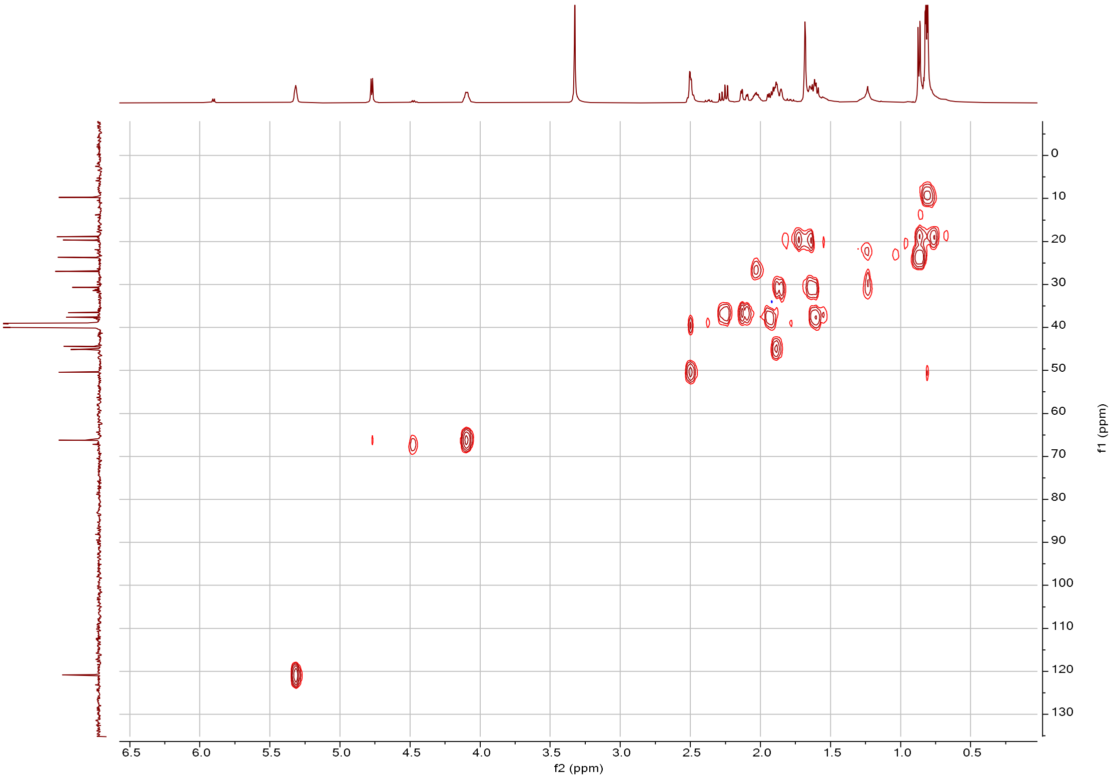

Figure S49. HMBC spectrum of compound **6**.

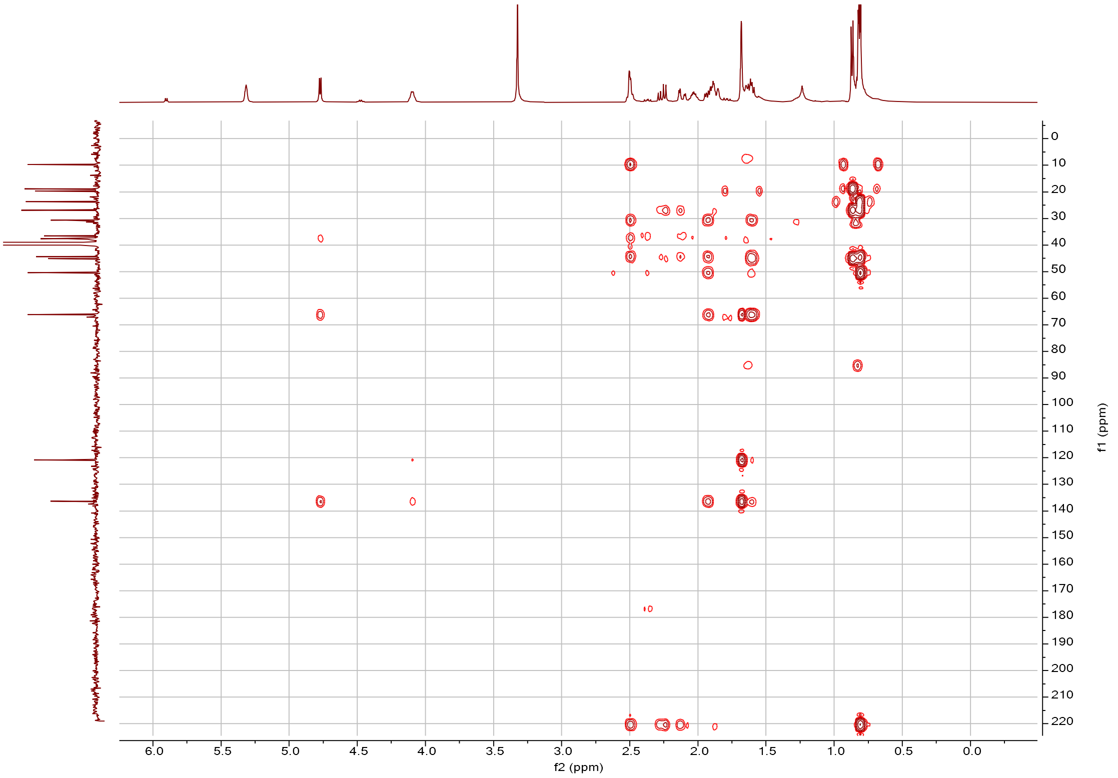

Figure S50. NOESY spectrum of compound **6**.

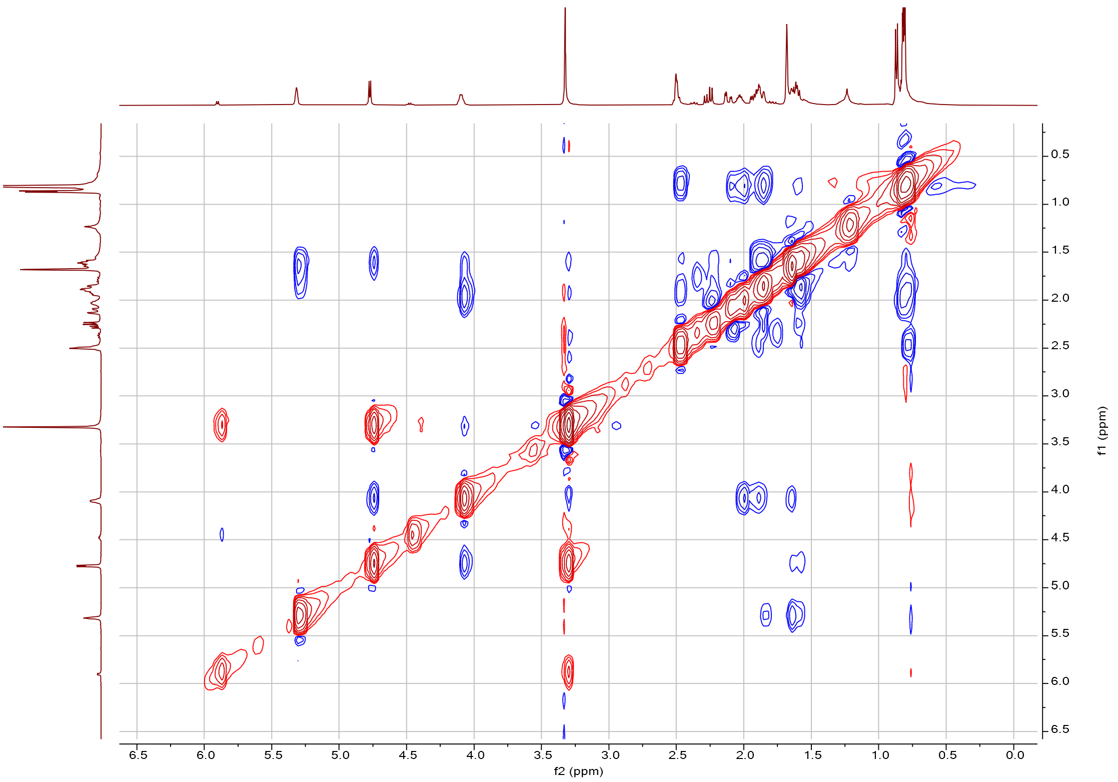

Figure S51. HRESI mass spectrum of compound **6**.

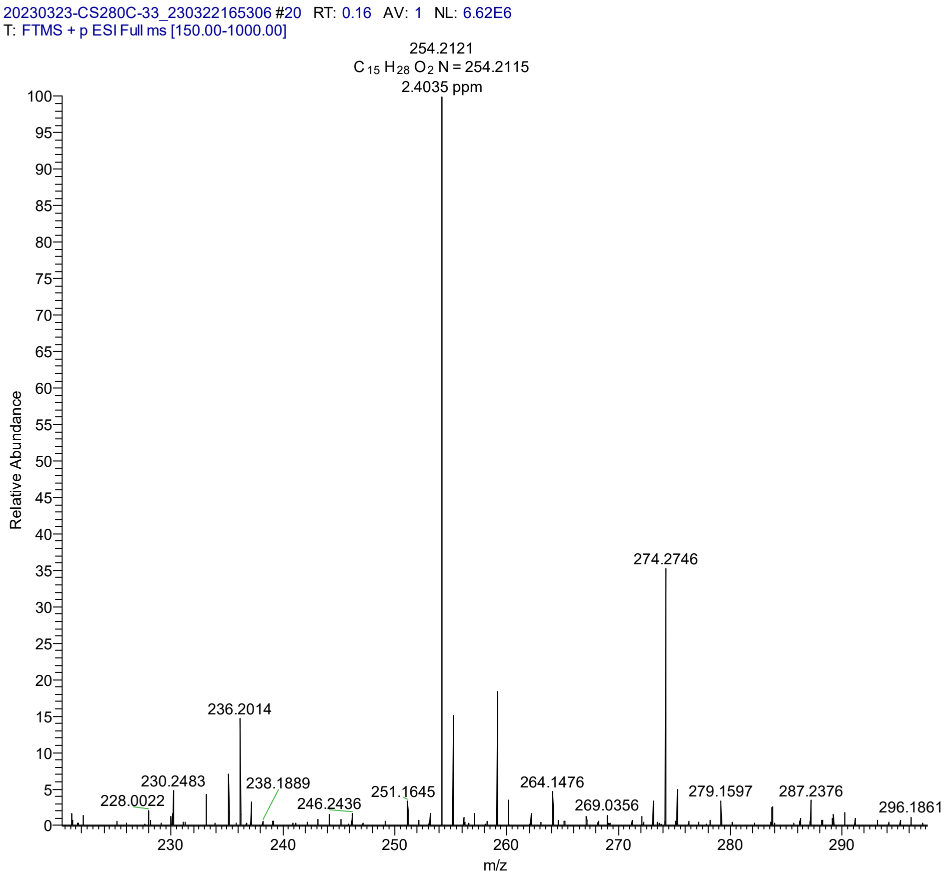

Figure S52. ECD spectrum of compound **6**.

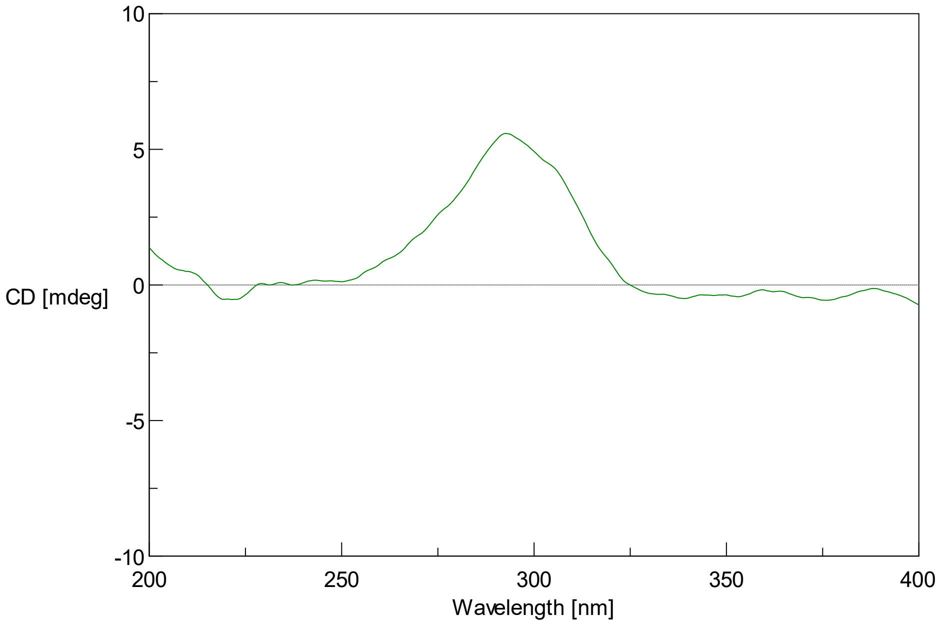

Figure S53. Chart flow of the isolation procedure.

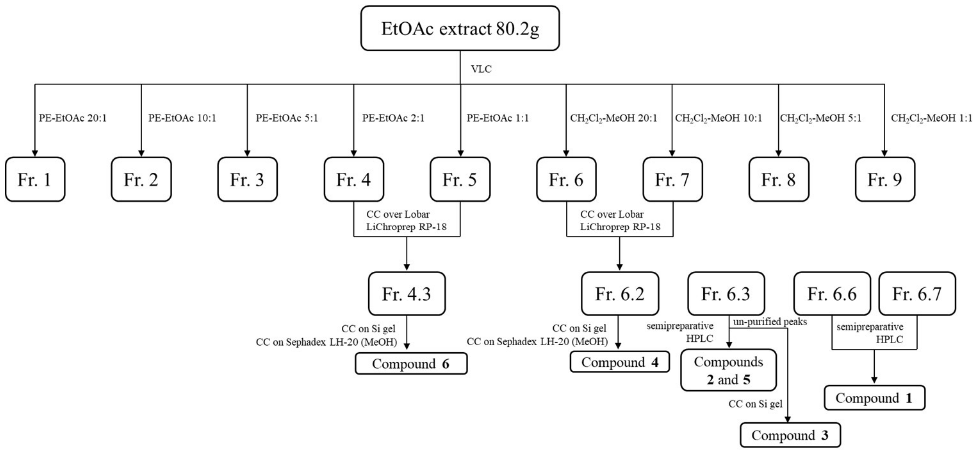

Figure S54. Proposed biogenetic network for compounds **1**–**6**.

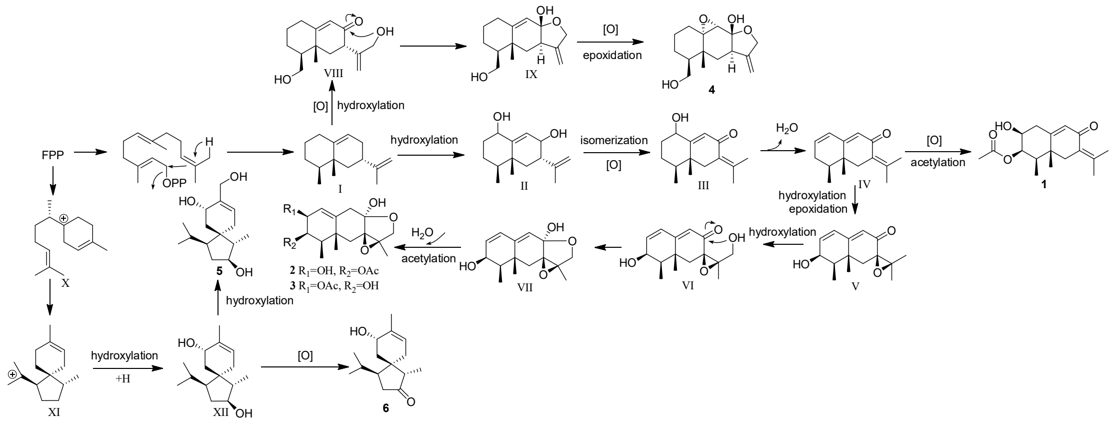

Supplement: Supplementary file 1 [file marinedrugs-22-00574-s001.zip › marinedrugs-3350436-supplementary.pdf]
